# Thermopower in Underpotential Deposition-Based Molecular Junctions

**DOI:** 10.1021/acs.nanolett.3c04438

**Published:** 2024-01-25

**Authors:** Peng He, Abdalghani H. S. Daaoub, Sara Sangtarash, Hatef Sadeghi, Hyo Jae Yoon

**Affiliations:** † Department of Chemistry, Korea University, Seoul 02841, Korea; ‡ Device Modelling Group, School of Engineering, University of Warwick, Coventry CV4 7AL, U.K.

**Keywords:** underpotential deposition (UPD), thermopower, molecular junction, tunneling, Seebeck coefficient

## Abstract

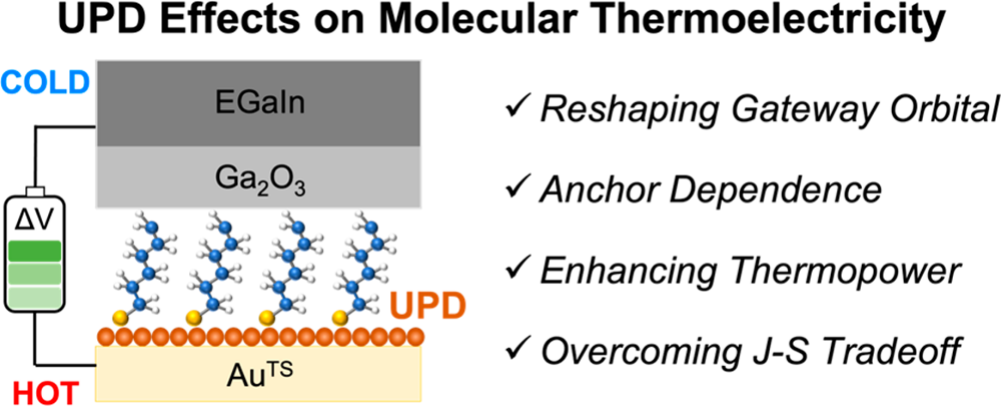

Underpotential deposition (UPD) is an intriguing means for tailoring the interfacial electronic structure of an adsorbate at a substrate. Here we investigate the impact of UPD on thermoelectricity occurring in molecular tunnel junctions based on alkyl self-assembled monolayers (SAMs). We observed noticeable enhancements in the Seebeck coefficient of alkanoic acid and alkanethiol monolayers, by up to 2- and 4-fold, respectively, upon replacement of a conventional Au electrode with an analogous bimetallic electrode, Cu UPD on Au. Quantum transport calculations indicated that the increased Seebeck coefficients are due to the UPD-induced changes in the shape or position of transmission resonances corresponding to gateway orbitals, which depend on the choice of the anchor group. Our work unveils UPD as a potent means for altering the shape of the tunneling energy barrier at the molecule–electrode contact of alkyl SAM-based junctions and hence enhancing thermoelectric performance.

Upon adsorption of a molecule onto a substrate, orbital mixing between molecule and the surface atom occurs that can lead to the creation of new in-gap energy states.^1−8^ In molecular electronics, understanding the nature of adsorption-induced new energy states is significantly important for investigating charge transport behavior and developing functionalities.^9−12^ Conventional electrical current measurements offer limited information about the energy topography of molecular-scale electronic devices.^13,14^ The Seebeck coefficient (*S*) is closely related to the shape of the transport resonance due to an accessible molecular orbital at the Fermi level.^15−25^ Generally, for molecular junctions where a single energy level governs charge transport, and there is a coupling between the molecule and electrodes, *S* can be estimated using the Mott formula (eq 1) combined with a Lorentzian-shaped transmission function [*T*(*E*)] (eq 2):^13,16,17,22,26^
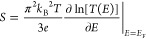
1
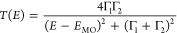
2where *E*_MO_ is the frontier molecular orbital energy, Γ_1_ and Γ_2_ are broadening of the molecular orbitals resulting from top and bottom contacts in a junction, respectively, *k*_B_ is the Boltzmann constant, *T* is the junction temperature, *e* is the electronic charge, and *E*_F_ is the Fermi level.

While molecular thermoelectrics has focused mainly on the molecular approach where the chemical structure of the active molecule is varied to control the energy offset (Δ*E*) between *E*_F_ and *E*_MO_ or Γ and enhance *S*,^17,20,22,27,28^ a handful of studies have adopted the nonmolecular approach, particularly with a focus on the identity of the electrode.^8,16,29−31^ Segalman observed variation of the *S* value of fullerene molecules as the top electrode of a single-molecule junction varied from gold to silver and platinum.^30^ This observation was attributed to the different Fermi levels of the metals, which leads to different Δ*E* values. Tada reported that the *S* value of benzenedithiol changed from 7.4 to −12.1 μV/K once the gold electrode was replaced with nickel.^31^ The change in the size and polarity of *S* was explained by the strong spin-split hybridization between the HOMO and the d band of nickel, which led to the change in Δ*E* and in the identity of the accessible orbital from the highest occupied molecular orbital (HOMO) to the lowest unoccupied molecular orbital (LUMO).

Beyond the monometallic materials, bimetallic materials—electrode materials consisting of two distinct metals in a layered structure or alloy forms—may provide insights into how to engineer the energy structure in molecular junctions. Underpotential deposition (UPD) is an electrochemical process in which a full monolayer of a foreign metal is deposited on a substrate at potentials lower than those predicted by the Nernst equation due to work function differences.^32^ This technique offers a potent means of creating bimetallic materials and investigating the impact of the adlayer of the second metal. Alternation of the electronic structure of the molecule–electrode interface through UPD has been utilized for modulating the adsorption affinity of a molecule,^33−35^ heterogeneous catalytic properties,^36−38^ and plasmonic properties.^39^ Recently, UPD-based bimetallic electrodes were employed to enhance the electrical conductance of junctions.^40,41^ Gu et al.^40^ observed improved conductance of α,ω-alkanoic acids by modifying the bare gold electrode with UPD adlayers of silver or copper. The conductance increased by 40–60-fold compared to that of the unmodified electrode, which was attributed to the smaller Δ*E* induced by a d-band shift and stronger coupling between the molecule and electrode.

Here we show how the UPD-based bimetallic electrode (BE) affects the thermopower of self-assembled monolayer (SAM)-based junctions (Figure 1a). We introduced an UPD Cu adlayer on the surface of template-stripped gold (Au^TS^) to produce the BE, Cu-UPD/Au^TS^. We formed SAMs on the BE and monometallic electrodes (MEs), Au^TS^ or Ag^TS^, using *n*-alkanethiols (HSC*_n_*; *n* = 4, 6, 8, 10, or 12) and *n*-alkanoic acids (HO_2_CC_*n*–1_; *n* = 8, 10, 12, or 14) (Figure 1b). Junction measurements with the eutectic Ga–In (EGaIn) technique (Figure 1a)^22,42,43^ revealed an *S* increased by ≤4-fold when the ME was replaced with the BE. First-principles quantum transport calculations suggested that the presence of the Cu UPD adlayer significantly altered the shape and/or position of transmission resonances corresponding to gateway orbitals (GWOs)^44,45^ near *E*_F_. This effect was strongly influenced by the anchor group, which addressed our findings.

**Figure 1:**
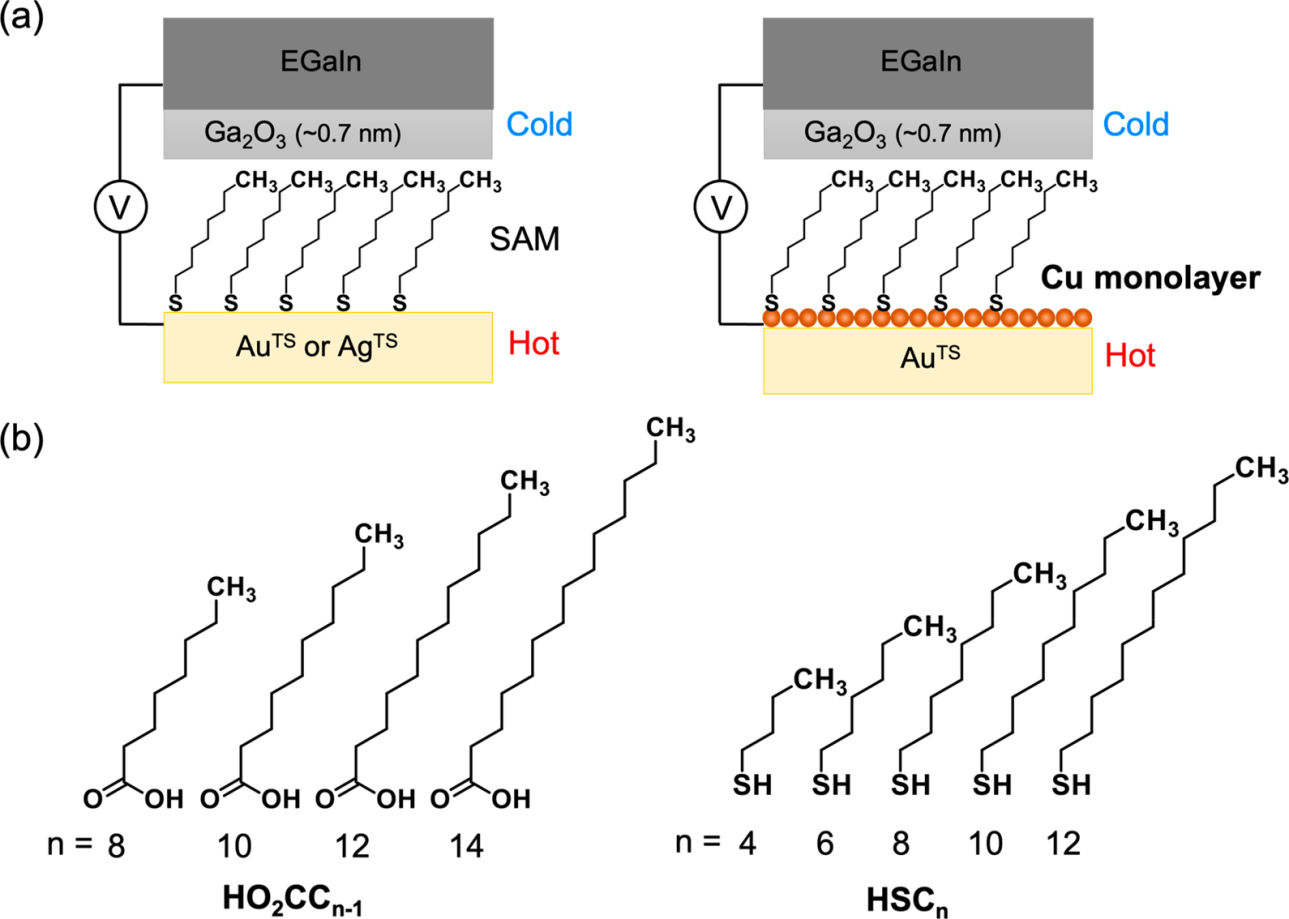
(a) Schematic describing the structure of the large-area junctions we used. Charge transport properties of self-assembled monolayers (SAMs) formed on monometallic (ME) and bimetallic (BE) electrodes were compared. The ME is either template-stripped gold (Au^TS^) or silver (Ag^TS^), and the BE is Au^TS^ covered with Cu monatomic adlayer via underpotential deposition (UPD) (EGaIn/Ga_2_O_3_, eutectic Ga–In covered by self-passivating oxide skin). (b) Alkyl derivatives used in this work. The molecules have thiol or carboxylic acid anchoring groups.

We focused on *n*-alkanethiols and *n*-alkanoic acids for the following reasons. (i) *n*-Alkanoic acids afford monolayers on both Cu-UPD/Au^TS^ BE and Ag^TS^ ME, while *n*-alkanethiols do on both Cu-UPD/Au^TS^ BE and Au^TS^ ME,^33,46−48^ allowing straightforward separation of the effect of UPD on thermopower. (ii) The Seebeck coefficients of their SAMs on the ME are known.^1,16^ (iii) Their transport mechanisms have been well-defined.^49,50^ For UPD, we followed the literature procedures.^46^Figure 2a shows cyclic voltammetry (CV) curves for UPD and overpotential deposition (OPD) of the Cu adlayer on the Au^TS^ substrate in a N_2_-saturated solution containing 1 mM CuSO_4_ and 0.1 M H_2_SO_4_. Distinct peaks corresponding to UPD (**A1** and **A2** for deposition and **D1** and **D2** for stripping) and OPD (**B1** and **C1** for deposition and stripping, respectively) were observed, consistent with the literature.^46^Figure 2b illustrates the deposition and stripping processes during reduction and oxidation, respectively. Separate control experiments at various voltages and through a linear sweeping voltage method further ensured the desired operation of UPD and the formation of a monatomic Cu adlayer (see the Supporting Information for details). The presence of the monatomic Cu adlayer was confirmed through X-ray photoelectron spectroscopy (XPS). The Cu 2p spectrum exhibited doublet peaks at 931.9 and 951.8 eV, corresponding to Cu 2p_3/2_ and Cu 2p_1/2_, respectively (Figure 2c). The binding energy of Cu 2p_3/2_ for the Cu adlayer on gold was lower by ∼0.7 eV than that (932.6 eV) of the bulk Cu, consistent with the literature involving Cu UPD.^47,51^ The Cu 2p region was indicative of Cu(0) or Cu(I) species.^38^ The relative atomic ratios of Cu and Au atoms were 15% and 85%, respectively, which were similar to the literature result.^38^

**Figure 2:**
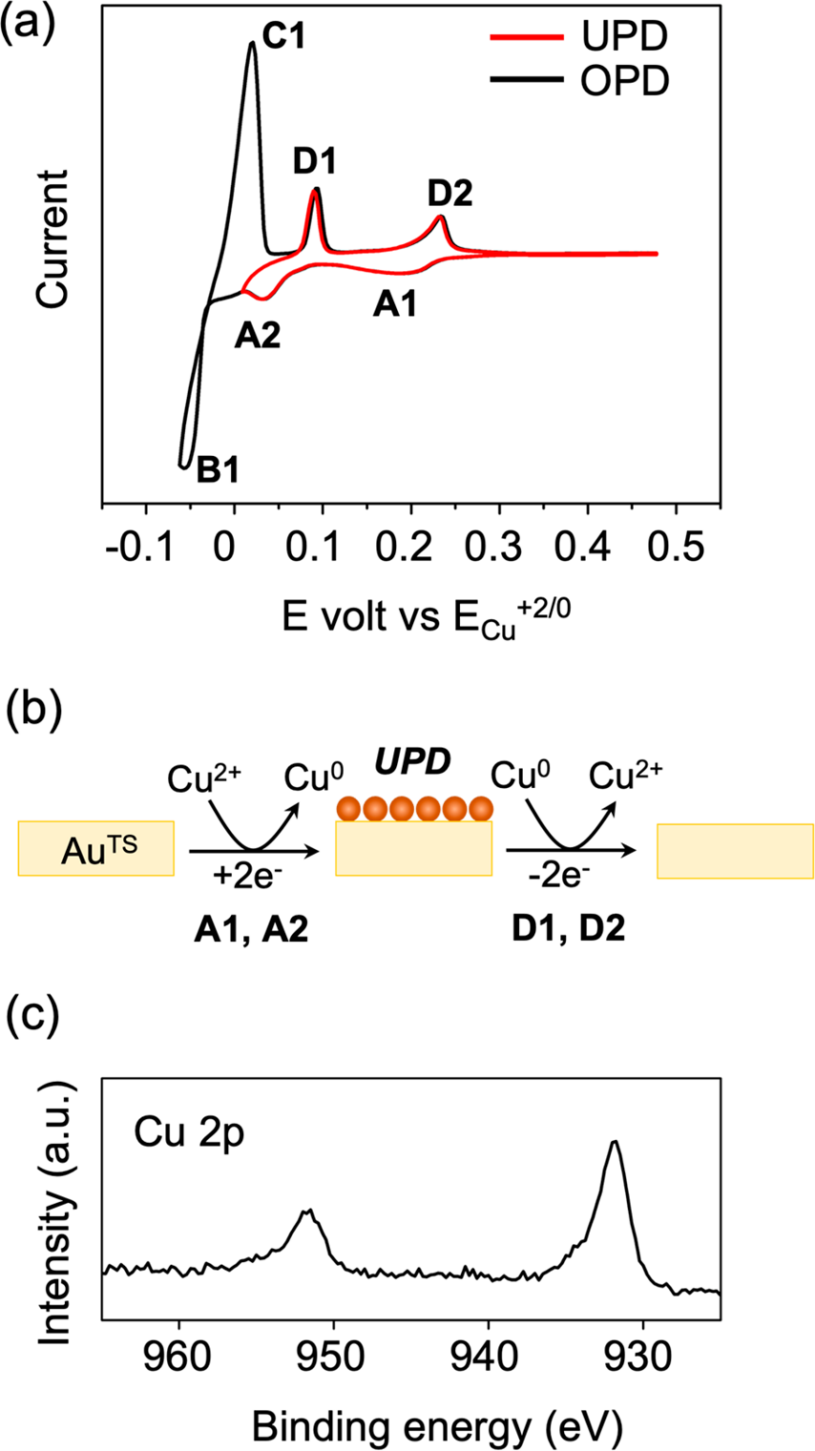
(a) Cyclic voltammograms of copper underpotential deposition (UPD) and overpotential deposition (OPD) on the Au^TS^ substrate. (b) Schematic describing transformation between a monometallic electrode, Au^TS^, and a bimetallic one, Cu/Au^TS^, via UPD. (c) High-resolution X-ray photoelectron spectra of Cu 2p for a Cu/Au^TS^ bimetallic electrode (BE). The separation between these two peaks was approximately 20.2 eV, consistent with the reported XPS result.^52^

We formed SAMs following the previously reported procedures.^33,47^ Detailed experimental procedures are described in the Supporting Information. The SAMs were characterized by XPS. After the formation of SAM on the BE, the binding energies of both Cu 2p_3/2_ and Cu 2p_1/2_ peaks were slightly shifted toward positive values (Figure S3), consistent with the literature result.^47^ For SC_8_ molecules, the S 2p signal (Figure S4) displayed a doublet peak observed at 162.1 and 163.3 eV, which were attributed to S 2p_3/2_ and 2p_1/2_, respectively. These binding energies were similar to those for the analogous SAMs on bulk copper substrates.^47,51,53^ In the HO_2_CC_7_ SAM on the BE, the C 1s XP spectrum exhibited two peaks at 284.5 and 287.3 eV assigned to the alkyl carbons and the bonding carboxylate group, respectively (Figure S4). No signal characteristic of free carboxylic acid (∼288.5 eV) was detected, indicating the formation of the desired SAM of alkanoic acid on the BE surface.^54,55^ A single binding energy of O 1s was observed at 531.3 eV (Figure S4), suggesting symmetric binding of the carboxylate group to the BE,^54,55^ similar to that on silver. Further discussion regarding the effect of the binding mode of -COOH on the thermopower and packing quality of the SAM on the UPD surface is provided in the Supporting Information.

Using the EGaIn technique,^16,22,42,56^ we formed junctions with the structure, substrate/SAM//Ga_2_O_3_/EGaIn (substrate = ME or BE; one slash and two slashes indicate covalent and van der Waals interfaces, respectively), and obtained thermovoltage (Δ*V*, microvolts) data at various temperature differentials (Δ*T* = 4, 8, 12, 15, or 20 K). At each Δ*T*, 1350–3075 Δ*V* data points were collected from 18–41 separate junctions in two different samples. Histograms of Δ*V* were generated, and mean (μ_Δ*V*_) and standard deviation (σ_Δ*V*_) values were obtained through single-Gaussian curve fitting (Figure 3a). The *S* values of SAMs were derived from plots of μ_Δ*V*_ versus Δ*T* (Figure 3b), following the previously reported procedures.^42^Figures S5 and S6 contain plots of all of the molecules tested; the data from the junction measurements are summarized in Tables S1 and S2. The yields of working junctions for the alkanoic acid and alkanethiolate SAMs were 77–100% and 83–100%, respectively.

**Figure 3:**
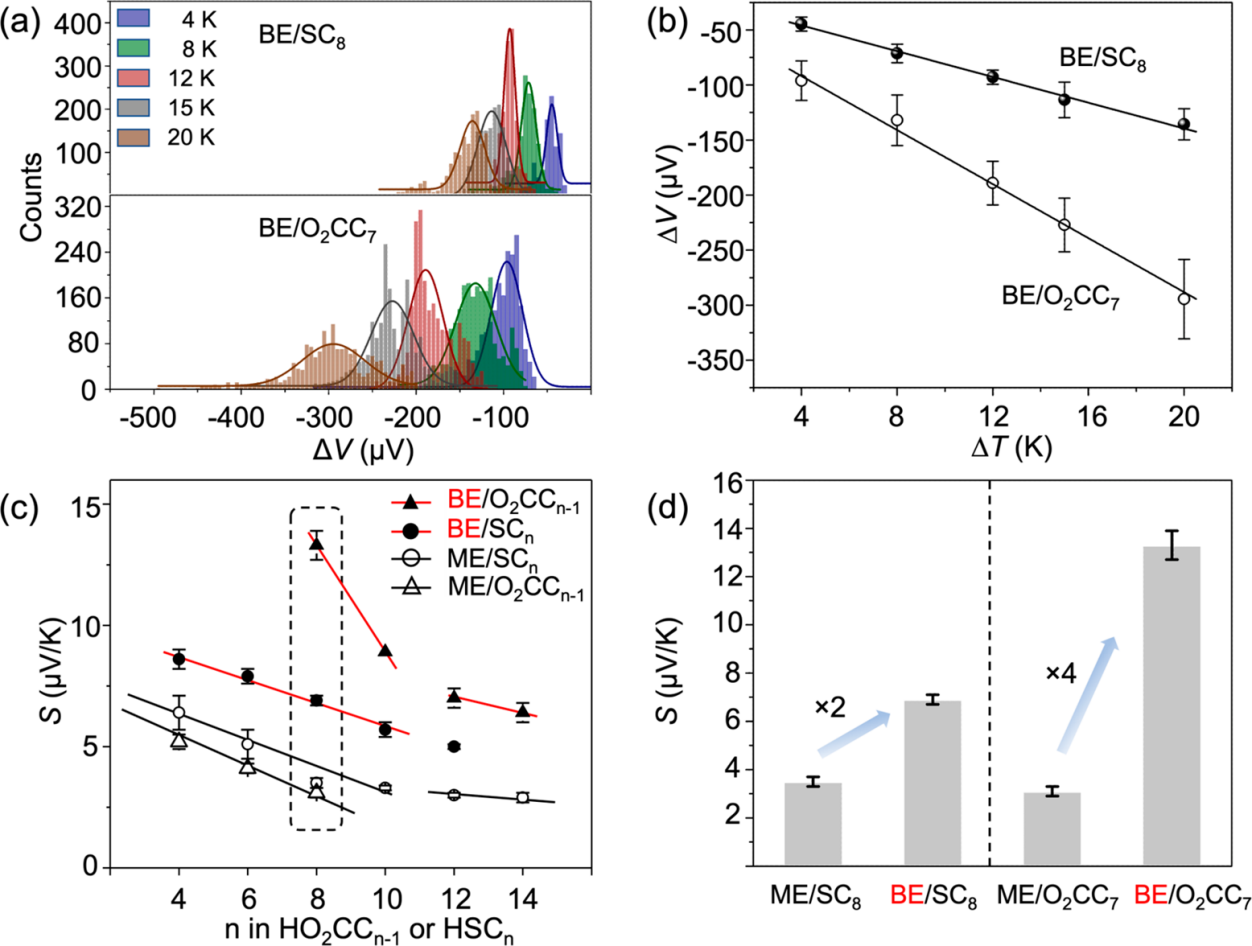
(a) Representative histograms of thermovoltage (Δ*V*, microvolts) of SC_*n*_ (*n* = 8) and O_2_CC_*n*–1_ (*n* = 8) SAMs on the BE at various temperature differentials (Δ*T*, kelvin). (b) Corresponding plots of Δ*V* as a function of Δ*V*. (c) Comparison of Seebeck coefficients (*S*, microvolts per kelvin) between the values of the O_2_CC_*n*–1_ and SC_*n*_ SAMs on the BE and the ME (Ag^TS^ and Au^TS^ for the O_2_CC_*n*–1_ and SC*_n_* SAMs, respectively). (d) Thermopower enhancements upon replacement of the ME with the BE.

All of the SAMs exhibited positive *S* values (Figure 3c), indicating that the HOMO dominates the transport (see below for details). The SAM of the alkyl backbone usually shows linear regression of *S* with an increase in the molecular length.^1,8^ We observed a similar trend in our SAMs, regardless of the type of electrode (ME vs BE) and anchor group (-CO_2_H vs -SH). The values of *S* in BE/O_2_CC_*n*–1_ and BE/SC_*n*_ ranged from 13.3 to 6.4 μV/K and from 8.6 to 5.0 μV/K, respectively. The *S* values on the BE were higher than those on the ME by up to ∼4.3 and ∼1.8 times for alkanoic acid and alkanethiolate SAMs, respectively (Figure 3d). The slope of the length dependence plot varied at the point near *n* = 10, similar to the trend of alkanethiolate SAMs on pure gold.^1^ We further discuss this below.

The work function of the Cu-UPD/Au substrate is 5.2 eV,^57^ which falls between those of pure gold (5.5 eV) and copper (4.6 eV). A lower work function would induce a smaller Δ*E* and thus decreased *S*.^19^ This prediction contrasts with our findings, prompting us to consider another mechanism. When alkyl molecules are adsorbed on metals, new in-gap states, called chemisorption-induced gap states^1,2^ or GWO,^45,58^ emerge. The new states largely depend on the identity and structure of the metal and anchor group.^59,60^ To investigate the interplay between the monatomic Cu adlayer and the new states, we conducted theoretical simulations using density functional theory (DFT) and quantum transport calculations. Initially, we found the molecular ground-state geometries both in the gas phase and between the electrodes, employing the SIESTA implementation of DFT.^61^ Subsequently, we obtained the mean-field Hamiltonian for each system based on DFT results, and we coupled it with the GOLLUM quantum transport code, to compute transmission coefficient *T*(*E*) for electrons of energy *E* as they traverse from one electrode to the other.^62,63^ See the Supporting Information for details of computational methods. In molecular junctions formed from a parallel array of molecules, different molecules might have different contact configurations with the electrodes and hence different *T*(*E*) values. In a SAM of *N* molecules in parallel, if molecule *j* has *T*_*j*_(*E*), the average *S* (*S̅*) is described as follows:
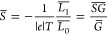
3where  and . In this expression, *f* = [e^(*E*–*E*_F_)/*k*_B_*T*^ + 1]^−1^ is the Fermi–Dirac probability distribution function and *T* is the temperature. From eq 3, both electrical conductance *G* and Seebeck coefficient *S* for each molecule with different electrode/molecule configurations need to be calculated to obtain the average *S* ( over a parallel array of molecules. Therefore, we constructed a series of junctions with different contact configurations, calculated *T*(*E*) for each junction, and used eq 3 to obtain *S̅*.

Figure 4a and Figures S7–S9 show the average *T*(*E*) for a set of structures consisting of alkanethiol wires sandwiched between two electrodes labeled as **1** (Au/SC*_n_*//Au) with different lengths (*n* = 2, ..., 18). It is clear from Figure 4b that *n*-alkanethiolates exhibit relatively wide energy gaps between their HOMO and LUMO, ∼9 eV, as reported previously.^64^ This also shows agreement with the previously reported position of HOMO and LUMO resonances at approximately −4.0 and 5.0 eV, respectively.^64^ The interaction between the contact sulfur atom in the molecule and the electrode atoms in the Au/alkanethiol//Au junction resulted in the formation of additional states within the HOMO–LUMO gap at −0.5 eV (black dashed line in Figure 4b) relative to the DFT Fermi energy (*E*_F_ = 0 eV). This state is primarily attributed to the presence of the sulfur atom and is termed GWO.^45^ To investigate the origin of this transmission feature, we conducted *T*(*E*) calculations for three different junctions: Au//C_4_//Au, Au/SC_4_//Au, and Au/SC_4_S/Au as shown in Figure S10. Clearly, in the presence of the terminal sulfur atom, a new transport resonance formed in the energy gap of the Au//C_4_//Au junction. Furthermore, our local density of state (LDOS) calculations around this resonance for all junctions with different lengths (Figures S11 and S12) showed that the charge density is localized on the terminal sulfur atom, confirming that the GWO due to this atom is responsible for the transport resonance close to *E*_F_.

**Figure 4:**
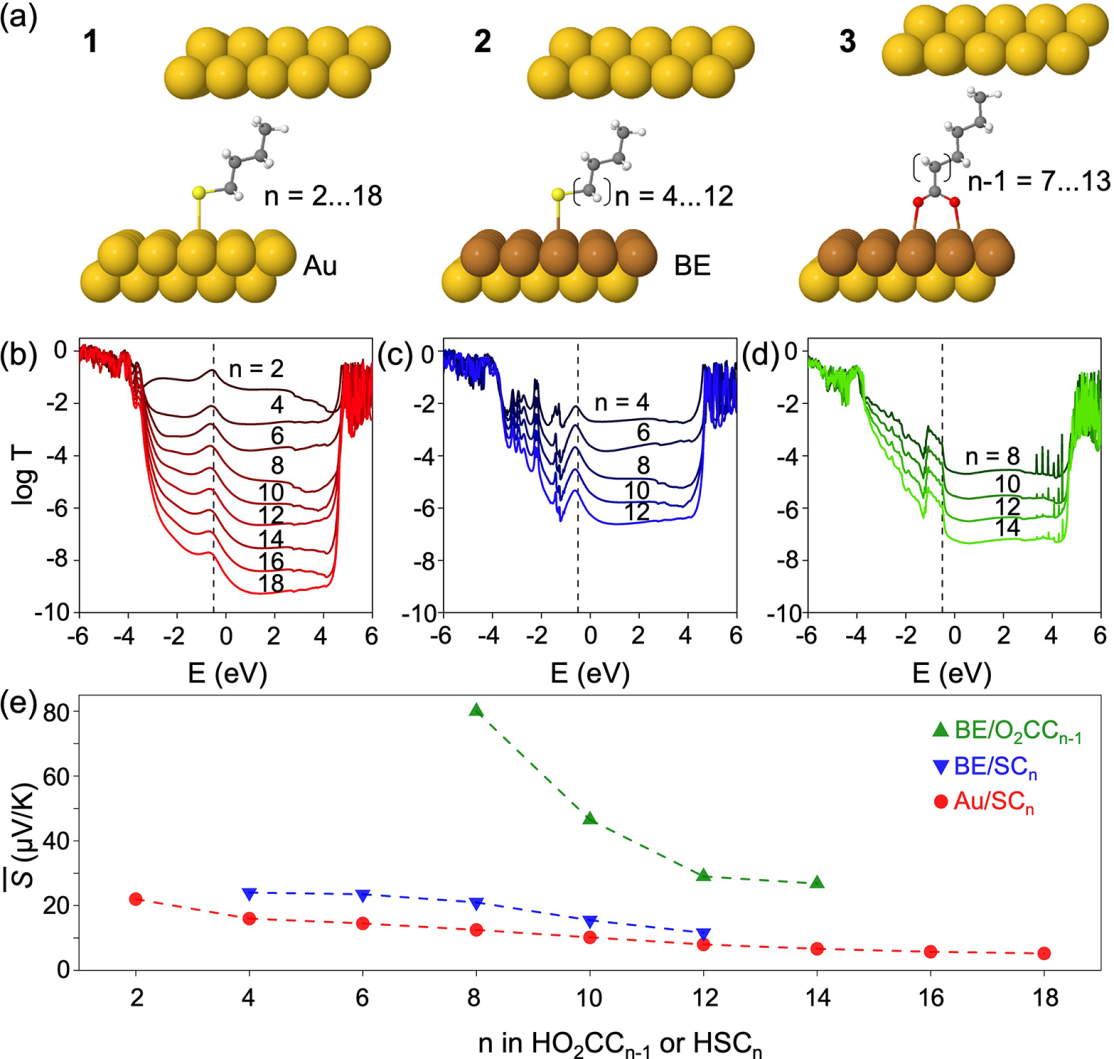
(a) Molecular junctions formed by alkanethiol between electrodes **1** (Au/SC*_n_*//Au), **2** (BE/SC_*n*_//Au), and alkane-carboxylic between electrodes **3** (BE/O_2_CC_*n*–1_//Au) with different lengths (*n*, the number of carbon in the alkyl backbone). Transmission coefficients for (b) **1**, (c) **2**, and (d) **3**. (e) Comparison of the average Seebeck coefficients for **1**–**3** at an *E*_F_ of 0.5 eV (black dashed line in panels b–d). *E* = 0 eV denotes the DFT Fermi energy in panels b–d.

From DFT *T*(*E*) values, we calculated the average *S* (*S̅*) for molecules with different lengths using eq 3. The *S̅* of alkanethiolate decreased with length (Figure S13) in agreement with our experiment and the previous reports.^1,65^ This is because the width of the GWO state increases with the length of the alkane chain. Because the Seebeck coefficient is proportional to the slope of *T*(*E*),^63^ this increase in the width of the GWO state with the length leads to a decrease in the slope of *T*(*E*) and hence reduces *S*.

There was a transition in the slope of the length dependence at *n* = 10 (Figure 3c).^1^ Our calculations showed that the resonance caused by the GWO states becomes broader as the junction length increases (see Figures S13, S15, and S16). This leads to a faster decrease in *S* with length when the resonance is sharp and a slower decrease when the resonance is broader. Additionally, the resonance moves closer to the Fermi energy as the molecular length increases. This further decreases the rate of the decrease in *S* with length. These two effects combined cause *S* to decrease at a faster rate initially, but the rate of the decrease slows for longer junctions, addressing the observation of two different length dependence regimes in the SC*_n_* SAMs on Au^TS^. Calculations to examine the intermolecular packing effect on *S* were conducted (Figure S14). The transmission plots for alkanethiol-based junctions with different intermolecular distances were nearly identical for a wide energy range around the Fermi level, indicating the insignificant role of intermolecular interaction in the thermopower of these molecules.

Next, we calculated the DFT *T*(*E*) values for junctions formed by the Cu-coated bottom gold electrode as shown in Figure 4c and Figure S15. Interestingly, the GWO states also formed in the BE/SC_*n*_//Au junctions. Our LDOS calculations for the energies around the energy of the GWO resonance (Figure S12) showed that the charge density is localized on both the terminal sulfur atom and Cu atoms, suggesting that this GWO state is due to the hybridization of the orbitals of the terminal sulfur atom and the Cu layer. The width of resonances due to this GWO state was smaller than that for the corresponding *n*-alkanethiolate with a similar length on the Au without a Cu adlayer. Consequently, the slope of *T*(*E*) on the Au/Cu BE was larger, leading to a higher *S̅* in BE/SC_*n*_//Au junctions than in Au/SC*_n_*//Au junctions as shown in Figures S13 and S15. For the carboxylic acid anchor system, the weaker interaction between -COO^–^ and the Cu layer resulted in a sharp slope of *T*(*E*) close to the GWO states (Figure 4d), leading to a high *S̅*. Figure 4e (and Figure S21) compares *S̅* values at an *E*_F_ of −0.5 eV (black dashed line in Figure 4b–d) for the different junctions shown in Figure 4a. The BE/O_2_CC_*n*–1_//Au junction exhibited the highest *S̅*, while the Au/SC*_n_*//Au junction showed the lowest *S̅*, in agreement with our experimental result in Figure 3c. Note that the DFT calculation indicated the possible effect of the binding modality of carboxylic acid on *S*. The GWO states in the binding with one oxygen were far from the Fermi energy compared to those in the binding with two oxygens (Figures S16–S20).

We verified the existence of the GWO state arising from the interaction between the two anchor groups and the BE through measurements of current density (*J*, amperes per square centimeter). The DFT calculations indicated that there is no significant difference in energy offset between the GWO and *E*_F_ in BE/SC*_n_* and gold ME/SC*_n_* systems, proposing that the conductance of the BE/SC*_n_* system would be on par with that of the ME/SC*_n_* system (Figure S22). To verify the result of the calculation, we obtained log|*J*|−voltage traces for the thiolate (HSC_8_) and alkanoic acid (HO_2_CC_7_) SAMs of an identical alkyl backbone on the BE and ME, respectively (Figure S24). As summarized in Table S7, log|*J*| values at 0.5 V for the SAM on the BE (0.51 ± 0.46 A/cm^2^) and the gold ME (0.58 ± 0.24 A/cm^2^) were indistinguishable from each other, proving the result of our calculation. In contrast to the result of the thiolate SAM, the log|*J*| value at 0.5 V for the O_2_CC_7_ SAM increased from −0.54 ± 0.95 to 0.73 ± 0.94 A/cm^2^ when the silver ME was replaced with the BE (Figure S24 and Table S7). The same trend was observed in the conductance results of our junctions (see the Supporting Information for details). These trends concur well with the results of DFT calculations (Figures S22 and S23). The simulation of transmission plots indicated that the enhanced *J* on the BE is due to the UPD-induced reduction of the energy offset between the GWO and *E*_F_. For the alkanoic acid, the *S* and *J* values both increased on the BE compared to those on the ME. This implies that the choice of the anchor group in combination with UPD can help avoid an important issue in molecular thermoelectricity, the trade-off between thermopower and conductance as a result of a structural change in a molecular junction.^28,56,66,67^ Indeed, power factor of the octanoic acid SAM increased by 3 orders of magnitude, from 3.4 × 10^–11^ to 1.1 × 10^–8^ μW m^–1^ K^–2^ upon replacement of the silver ME with the BE^56^ (see the Supporting Information for details). Calculations revealed no significant difference in the tunneling attenuation coefficient for the junctions with and without the Cu UPD layer, consistent with the literature.^40^

In summary, our study investigated the impact of UPD on the thermoelectric properties of alkyl SAM-based junctions. By introducing a monatomic Cu adlayer via UPD on the Au^TS^ substrate, we observed noticeable enhancements, up to 4-fold, in *S*. DFT calculations revealed that the resonance peak of the GWO stemming from the hybridization between the anchor group and Cu adlayer orbitals plays a pivotal role in the enhancements of the thermopower of the molecular junctions, and this effect largely depends on the choice of the anchor group (thiol vs carboxylic acid). Our work advances our understanding of the interfacial energy topography in UPD-modified surfaces, demonstrating a strategy for tuning the thermoelectric effect of a nanoscale device.

## Supporting Information Available

Experimental details for the procedure of UPD, electrochemical measurements, SAM preparation, and junction measurements; details of computational and power factor calculations; discussion of the mode of binding of carboxylic acid on a Cu adlayer, quality of packing of the SAM on Cu adlayers, and effect of UPD on the conductance and tunneling attenuation coefficient; additional data and figures, including characterization of the UPD layer, XPS spectra, AFM measurements, thermovoltage histograms, and current density and conductance histograms; and additional computational results (PDF)
