# Supplementary material for: Thermopower in Underpotential Deposition-Based Molecular Junctions
Source: Nano Lett. 2024 Jan 25;24(6):1988–95. doi: 10.1021/acs.nanolett.3c04438 (PMC10870761; doi:10.1021/acs.nanolett.3c04438)
Supplement: Supplementary file 1 — nl3c04438_si_001.pdf [file nl3c04438_si_001.pdf]

Supporting Information

Thermopower in Underpotential Deposition-based Molecular Junctions

Peng He,[a ‡] Abdalghani H.S. Daaoub,[b ‡] Sara Sangtarash,[b] Hatef Sadeghi,[b*] and Hyo Jae Yoon[a*]

[a] Department of Chemistry, Korea University, Seoul, 02841, Korea

[b] School of Engineering, University of Warwick, Coventry CV4 7AL, UK

[‡] These authors contributed equally to this work.

*Corresponding Authors' Email: Hatef.Sadeghi@warwick.ac.uk (H.S.)

hyoon@korea.ac.kr (H.J.Y.)

Table of Contents

1. Experimental Details -- S3

1.1 Materials and Characterization --S3

1.2 Typical Experimental Procedure of Underpotential Deposition (UPD) ---S4

1.3 Electrochemical Characterization of UPD Layer ----------------------------S4

1.4 SAMs Preparation -- S5

1.5 Eutectic Gallium–Indium (EGaIn) System ----------------------------------- S5

1.6 DFT methods -- S6

1.7 Minor Discussions --- S7

2. Supplementary Figures and Tables --- S11

3. References -- S44

1. Experimental Details

1.1 Materials and Characterization

All organic solvents and reagents were purchased from TCI, Alfa Aesar, Daejung and Sigma-Aldrich. n-Alkanethiols (HSC_n; n = 4, 6, 8, 9, 10, 12; purity 95 – 99 %) were purchased from Sigma-Aldrich and TCI. n-Alkanoic acids (HO_2CC_{n-1}; n = 8, 10, 12, 14; purity 97 – 99%) were purchased from Sigma-Aldrich. High purity eutectic gallium-indium (EGaIn; 99.99%) was obtained from Sigma-Aldrich and used as supplied. Gold thin films (300 nm) were deposited onto silicon wafer (100 mm in diameter; 1 – 10 ohm-cm, 525 ± 50 microns thick) using an e-beam evaporator (ULVAC). Glass with a thickness of 1.2 – 1.5 mm used to prepare the template-stripped gold substrates was purchased from Matsunami and cut into 1 cm × 1 cm pieces. Optical adhesive was purchased from Norland (NOA81) and used as supplied.

All X-ray photoelectron spectroscopy (XPS) measurements were carried out on a Thermo Scientific Nexsa photoelectron spectrometer with a monochromated Al Kα X-ray source (1486.6 eV).

Atomic Force Microscopy (AFM) measurements were performed using a Bruker Multimode 8 instrument to assess the surface roughness of Au^{TS} both before and after the UPD process. SNL-10 AFM tips from Bruker, characterized by a resonant frequency of 65 kHz and a spring constant of 0.350 N/m, were utilized for the roughness measurements. During the scanning process, the set point was fixed at 1 V, and the scan rate was set to 1.32 Hz.

1.2 Typical Experimental Procedure of Underpotential Deposition (UPD)

The UPD was conducted following the previously reported procedures.[1, 2] In a typical experiment, a three-electrode system was utilized, consisting of a freshly prepared template-stripped gold (Au^{TS}), copper wire, and platinum wire as the working, reference, and counter electrodes, respectively. Electrodeposition was conducted in a N_2-saturated solution containing 1 mM $CuSO_4$ and 0.1 M H_2SO_4(aq).[1-3] Prior to use, the copper wire was polished with sandpaper and briefly immersed in diluted nitric acid to clean its surface. The potential was held at a value just positive of the bulk deposition peak (approximately 0.02 V vs. $Cu^{+2/0}$) over 60 s. The obtained bimetallic electrode was then removed from solution under potential control, rinsed with copious ethanol and dried by N_2 flow.

1.3 Electrochemical Characterization of UPD Layer

The formation of UPD layer was confirmed by electrochemical analysis, following the previously reported procedures.[1, 3, 4] To characterize the Cu UPD region, the same conditions as mentioned above were utilized. Cyclic voltammetry (CV) tests were conducted in the potential range of -0.07 to 0.5 V (vs. $Cu^{+2/0}$) at a scan rate of 0.05 V/s. For verifying the exact UPD window, potentiostatic conditions were employed with various applied potentials (-0.02, -0.01, 0, +0.01 and +0.02 V) for a duration of 60 seconds at room temperature. Subsequently, Cu stripping was carried out using a linear sweeping voltammetry (LSV) method in the same solution, where the potential was swept from the deposition potential to 0.48 V at a scan rate of 0.01 V/s.[4]

1.4 SAMs Preparation

We prepared SAMs following the procedure reported previously.[2,5] After the UPD process, the UPD-modified substrates were immediately transferred into solutions containing acid or thiol derivatives (1 mM; ethanol and n-hexadecane for alkanethiols and alkanoic acids, respectively). All samples were incubated in the solution under N_2 atmosphere for at least 12 h. The resulting SAMs of alkanethiols and alkanoic acids were rinsed with ethanol and hexane, respectively. All samples were dried with gentle blowing of N_2 prior to characterization.

1.5 Eutectic Gallium–Indium (EGaIn) System

The EGaIn based method for molecular thermopower measurement allows easy and convenient collection of large amounts of data for SAMs.[6] The soft liquid metal, covered by a spontaneously formed and conductive Ga_2O_3 layer with a nominal thickness of approximately 1 nm, represents a good choice for the top electrode in molecular junctions.[7] The reversibility, non-invasiveness, and well-defined interface of EGaIn-based junction technique enable the creation of a high yield of thermoelectric junctions.[8] The process for forming the junction and measuring thermopower followed previously reported procedures.[8] In brief, we applied five different temperature gradients (ΔT = 4, 8, 12, 15, 20 K) across the SAM and recorded the output voltage at each temperature. We gathered 1350 – 3075 ΔV data from 18 – 41 separate junctions in two different samples for each ΔT.

1.6 DFT methods

The optimized geometry and ground state Hamiltonian and overlap matrix elements of each structure were self-consistently obtained using the SIESTA implementation of density functional theory (DFT).[9] SIESTA employs norm-conserving pseudo-potentials to account for the core electrons and linear combinations of atomic orbitals to construct the valence states. The local density approximation (GGA) of the exchange and correlation functional is used with PBE parameterization, a double-ζ polarized (DZP) basis set, a real-space grid defined with an equivalent energy cut-off of 250 Ry. The geometry optimization for each structure is performed to the forces smaller than 10 meV/Å.

The mean-field Hamiltonian obtained from the converged DFT calculation was combined with the GOLLUM implementation of the non-equilibrium Green's function method to calculate the phase-coherent, elastic scattering properties of the each system consist of left gold (source) and right gold (drain) leads and the scattering region.[10, 11] The transmission coefficient *T(E)* for electrons of energy *E* (passing from the source to the drain) is calculated via the relation: $T(E) = Trace(\Gamma_R(E)G^R(E)\Gamma_L(E)G^{R\dagger}(E))$. In this expression, $\Gamma_{L,R}(E) = i\left(\sum_{L,R}(E) - \sum_{L,R}{}^{\dagger}(E)\right)$ describe the level broadening due to the coupling between left (L) and right (R) electrodes and the central scattering region, $\sum_{L,R}(E)$ are the retarded self-energies associated with this coupling and $G^R = (ES - H - \sum_L - \sum_R)^{-1}$ is the retarded Green's function.

Thermoelectric properties. Using the approach explained in previous work,[11] the electrical conductance $G = G_0L_0$ and the Seebeck coefficient $S = -\overline{L_1}/eT\overline{L_0}$ are

calculated from the electron transmission coefficient $T_j(E)$ where $\overline{L_n} = \sum_{j=1}^{N} L_n^j$ and $L_n^j = \int_{-\infty}^{+\infty} dE\ (E - E_F)^n\ T_j(E)(-\partial f(E,T,E_F)/\partial E)$ and $f = (e^{(E-E_F)/k_BT} + 1)^{-1}$ is the Fermi-Dirac probability distribution function, T is the temperature, E_F is the Fermi energy, $G_0 = 2e^2/h$ is the conductance quantum, e is electron charge and h is the Planck's constant.

1.7. Minor Discussions

Characterization of UPD. We conducted separate control experiments to ensure the desired operation of UPD and the formation of monoatomic Cu adlayer. First, we obtained a CV curve in the electrochemical condition without the Cu^{2+} ions (**Figure S1a** in the Supporting Information); no peaks were observed, confirming that the peaks in the **Figure 2a** originated from the reduction and oxidation of copper ions.[3] Second, we conducted electrochemical experiments at different voltages to determine the suitable threshold voltage for the UPD and bulk deposition.[4] The Au^{TS} substates were potentiostatted at several voltages (-0.02, -0.01, 0, +0.01 and +0.02 V) for 60 seconds, and the linear sweeping voltages (LSV) method was followed to remove all copper deposited on the gold surface. In the UPD region, the stripping charge should be independent of the parking time at the deposition potential.[4] The LSV curves indicated the bulk deposition occurred when the potential was more negative than 0 V (**Figure S1b**), and our UPD condition of +0.02 V was relevant to the creation of the monoatomic adlayer avoiding the bulk deposition. This deposition potential was further supported by the constant stripping current with different deposition time (**Figure S1c**).[4]

Surface Topography of Cu Adlayer. We determined whether the surface roughness varied significantly upon the Cu UPD using atomic force microscopy (AFM).

The use of template-stripped metal substrate is justified by the creation of ultraflat surface, which helps avoid creation of significant defects caused by a rough surface.[12] There was no significant difference in the surface roughness between Au^{TS} ME (rms = 0.29 ± 0.07 nm) and Cu/Au^{TS} BE (rms = 0.28 ± 0.12 nm) (**Figure S2**). This finding indicates homogeneous large-area deposition of monoatomic Cu adlayer rather than bulk or cluster depositions.

Binding Mode of Carboxylic Acid on Cu Adlayer. On bulk copper, a carboxylate group binds asymmetrically—only one oxygen binds to the surface—while the Cu monolayer formed by the UPD prefers the symmetric binding.[5, 13] This distinct binding structure of the carboxylate anchor group is attributed to the difference in the extent of surface oxide between the substrates.[14] The monoatomic Cu adlayer formed on gold via UPD displays significantly greater resistance against oxidation than the bulk Cu because oxidation on the bimetallic electrode is limited to a maximum of one layer of copper.[15]

We calculated the DFT $T(E)$s for the alkanoic acid junctions with two different contacting modalities (**Figure S16-17**): case 1 when only one oxygen atom is connected to Cu layer, and case 2 when both oxygen atoms are connected to Cu adlayer. The GWO state is due to the hybridized orbitals of both -C(=O)O^- and Cu in case 2, while it is due to the Cu adlayer only in case 1 as confirmed by our LDOS calculations in **Figures S18** and **S19**. The resonance due to the GWO states in case 1 is far from the Fermi energy and does not significantly contribute to S (**Figure S20**). In case 2, we found that the GWO state moves toward E_F (**Figure S18**). Also, the weaker interaction between C(=O)O- and the Cu layer resulted in a sharp slope of $T(E)$ close to the GWO states (**Figure 4d**) leading to a higher $\bar{S}$. **Figure 4e and S21** show the comparison between

$\bar{S}$ at E_F = -0.5 eV (black dashed line in **Figures 4b-d**) for different junctions shown in **Figure 4a**. The junction BE/O_2CC_{n-1}//Au exhibited the highest $\bar{S}$, while Au/SC_n//Au showed the lowest $\bar{S}$ in agreement with our experimental result in **Figure 3c**.

Packing Quality of SAMs on UPD surface. It has been reported that the packing quality of alkanethiol and alkanoic acid SAMs on the Cu UPD surface is not significantly different from those of the analogous SAMs on pure gold or silver.[1, 5, 14] We also observed the similar results. Static contact angles of decanoic acid SAMs on the Cu UPD (102 ± 4°) and Ag^{TS} (110 ± 1°) were indistinguishable; there was also no significant difference in water contact angle for 1-decanethiolate SAMs on the Cu UPD (111±2°) and Au^{TS} (115±4°).

Higher Simulated *S* Values Than Experimental Ones. We note that our simulations predicted a higher value of *S* than was observed in the experiments, even though the overall trend of the data was similar. This is likely due to the fact that the simulations assumed that the molecular junctions were perfectly stretched and clean, while in reality there are defects in the SAM and some molecules may not be fully stretched. These factors can all affect the shape of the transmission function and broaden the resonance near E_F, which can effectively lower the absolute value of *S*.[16]

Conductance. We further estimated conductance of our junctions following previously reported procedure.[17] The log|*G/A*| values at 0 V for octanethiolate SAMs on the BE (0.48 S/cm^2) and the gold ME (0.71 S/cm^2) were indistinguishable from each other (**Figure S24** and **Table S7**). Here, *G* is the conductance of a junction and *A* is the geometrical contact area (cm^2). In contrast, the log|*G/A*| values at 0 V for octanoic acid SAM increased from -0.38 to 0.85 S/cm^2, when the silver ME was replaced with the BE (**Figure S24** and **Table S7**). These trends concur well with the results of DFT

calculations (**Figure S22-23**).

Power Factor (PF). We followed the previously reported procedure to calculate the PF of the O_2CC_7 SAM on both BE and ME.[18] Generally, PF is determined by PF = $\sigma \times S^2$, where σ (μS cm^{-1}) represents electrical conductivity, and S (μV K^{-1}) represents the Seebeck coefficient. In this case, we obtained the σ values for BE/O_2CC_7 and ME/O_2CC_7 as 6.2×10^{-1} and 3.5×10^{-2} μS cm^{-1}. For the distance between two electrodes (d), we used the thickness value of HO_2CC_7 SAM on pure Ag (0.83 nm, reported by Tao[13]) and assumed that the thickness of the SAM on both ME and BE is similar for the sake of simplicity.[18] Consequently, PF values for BE/O_2CC_7 and ME/O_2CC_7 were revealed to be ~1.1×10^{-8} and ~3.4×10^{-11} μW m^{-1} K^{-2}, respectively.

UPD Does Not Impact the Tunneling Attenuation Coefficient (β). It has been reported that there is no significant difference in β for alkanoic acid on copper UPD (0.92 per carbon) and pure silver (0.95 per carbon).[19] We have calculated β values of our molecules (**Figures S25 and S26**) and found that the β values are consistent with the previous report[19] and do not vary significantly according to the presence or absence of Cu UPD layer (red and blue curves in **Figure S26**). Note that the shape, amplitude, and the position of the transmission resonance due to the GWO change by the alkyl chain length. Altogether, these lead to non-linear decrease of S as a function of the length which is also supported by our first-principle calculations in **Figure 4**.

2. Supplementary Figures and Tables

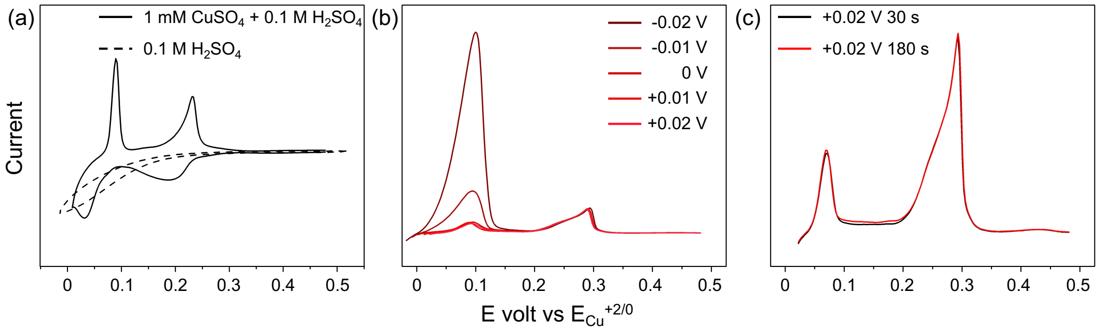

Figure S1. (a) CV of Au^{TS} in 0.1 M H_2SO_4 solution with or without 1 mM $CuSO_4$. (b) LSV of Cu/Au^{TS} prepared at different deposition potentials; scan rate is 1 mV s^{-1}. (c) LSV of Au^{TS} deposited for 30 s or 180 s under 0.02 V.

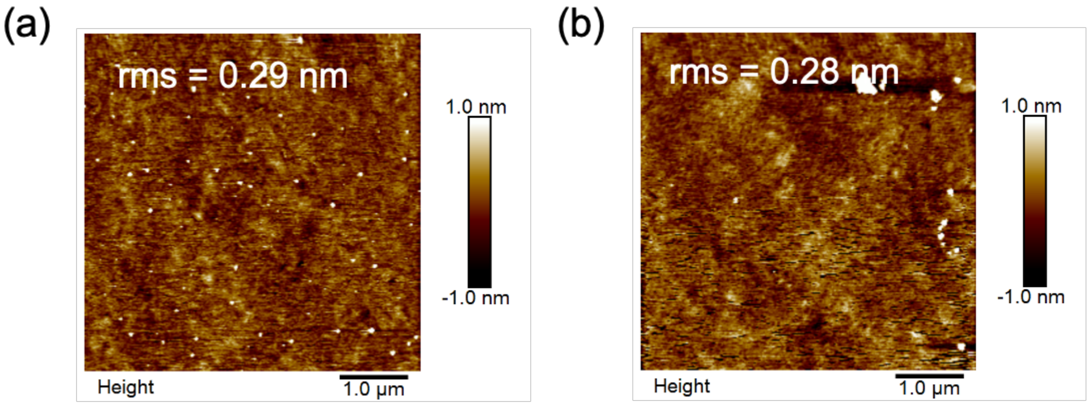

Figure S2. AFM analysis of (a) Au^{TS} and (b) Cu/ Au^{TS}.

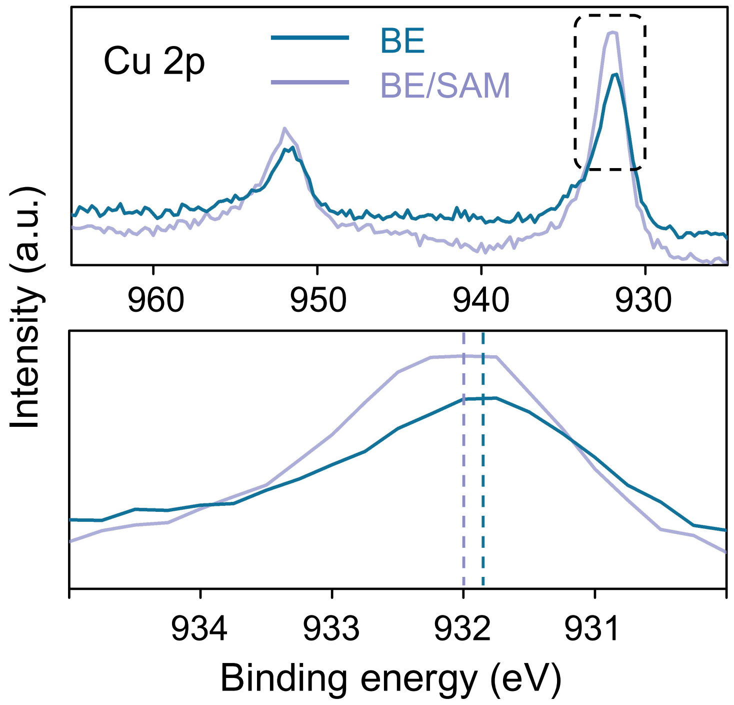

Figure S3. High-resolution X-ray photoelectron spectra of Cu 2p for Cu/Au^{TS} bimetallic electrode (BE) before and after the formation of SC_8 SAM.

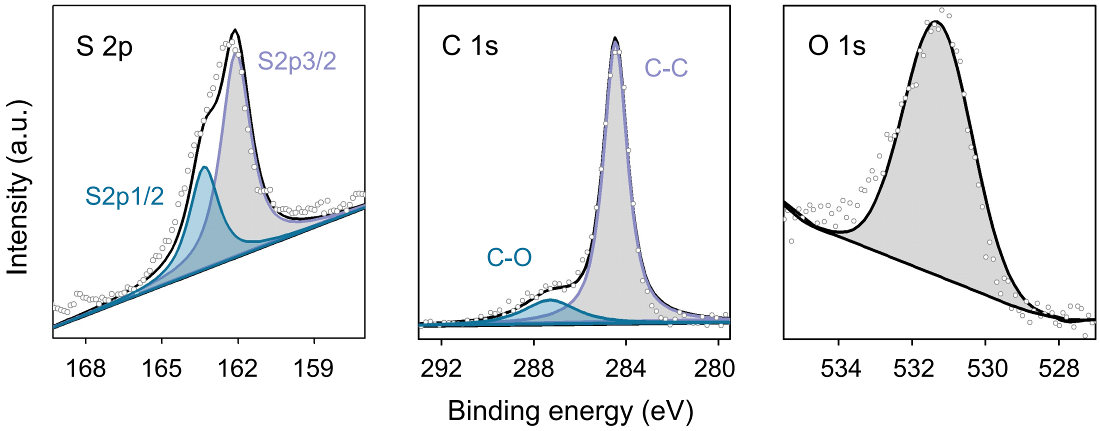

Figure S4. High-resolution X-ray photoelectron spectra of S 2p for BE/SC_8 SAM and of C 1s and O 1s for BE/O_2CC_7 SAM.

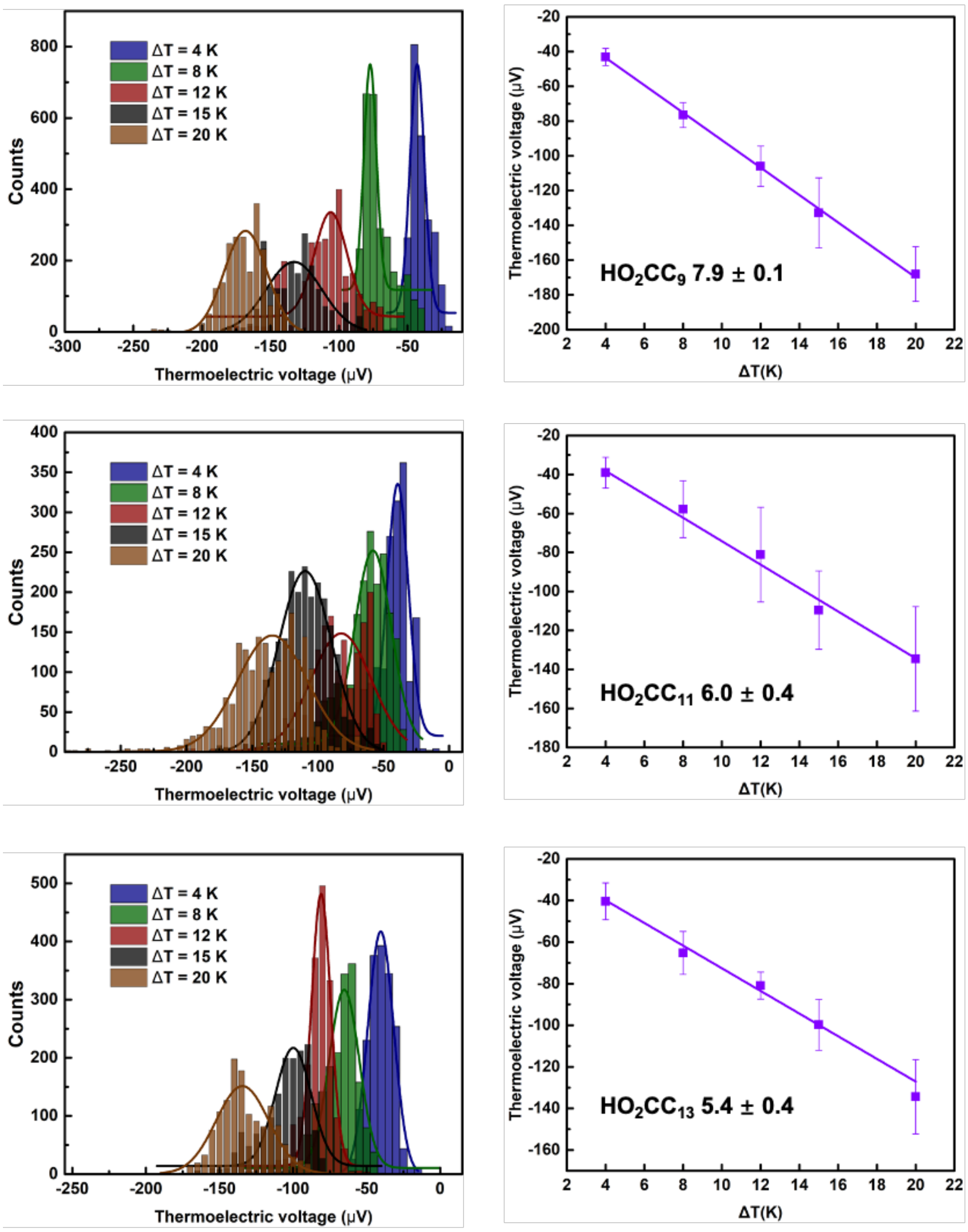

Figure S5. Histograms of thermoelectric voltage measured on BE/O_2CC_{n-1}//Ga_2O_3/EGaIn junctions.

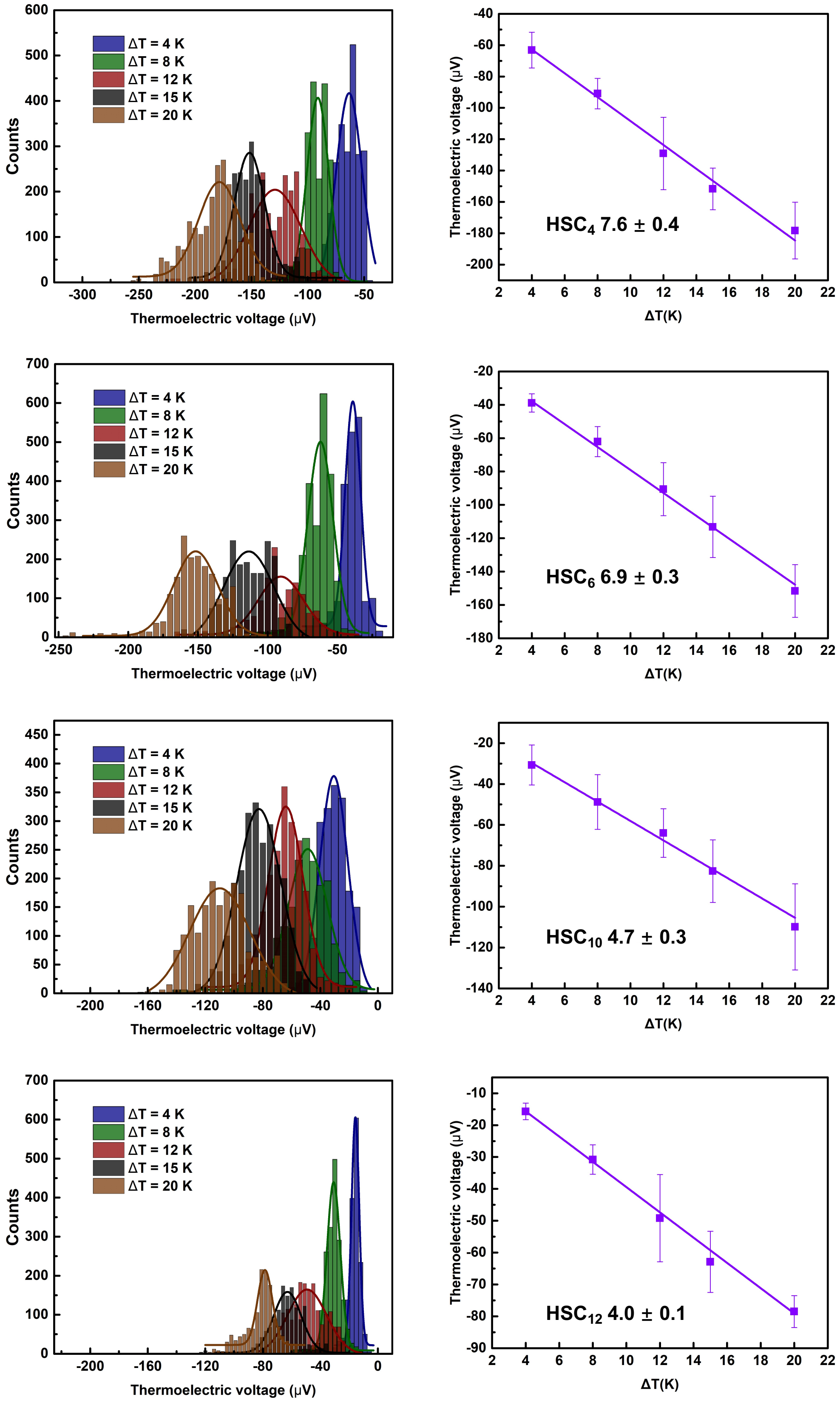

Figure S6. Histograms of thermoelectric voltage measured on BE/SC_n//Ga_2O_3/EGaIn junctions.

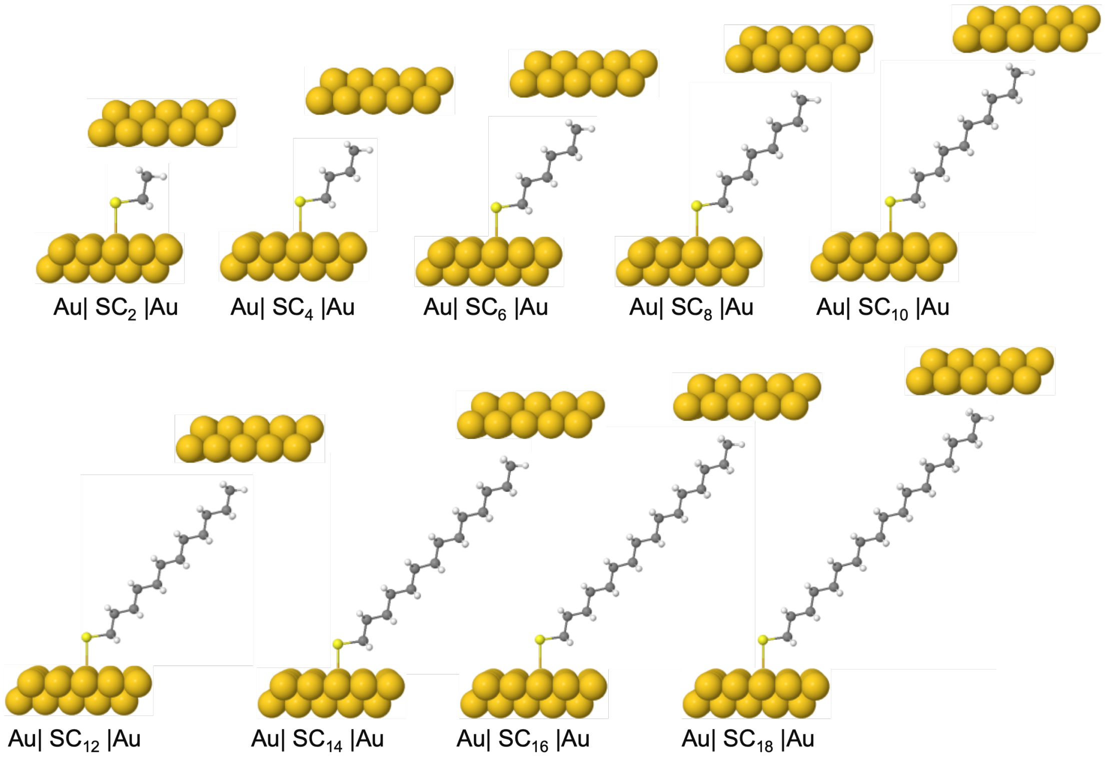

Figure S7. Molecular structures of alkanethiol wires sandwiched between two electrodes (Au/SC_n//Au), across a range of values for n (n = 2, ... ,18).

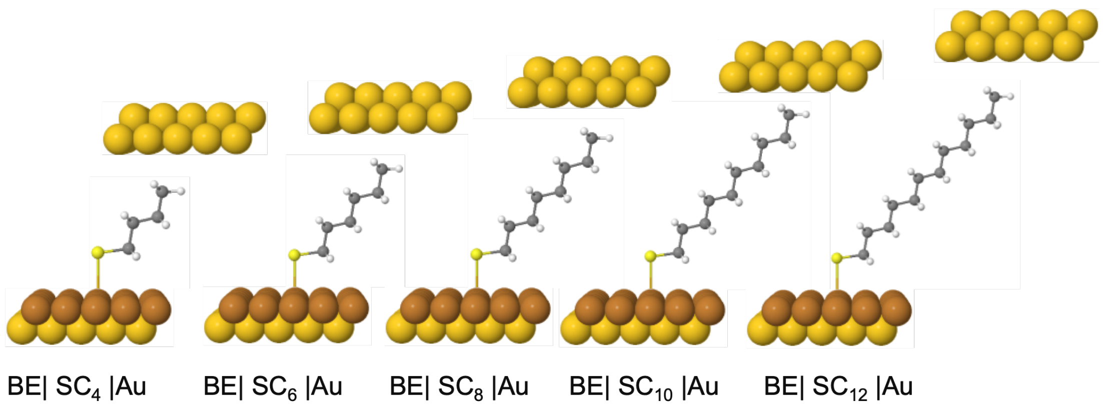

Figure S8. Molecular structures of alkanethiol wires sandwiched between two electrodes (BE/SC_n//Au), across a range of values for n (n = 4, ..., 12).

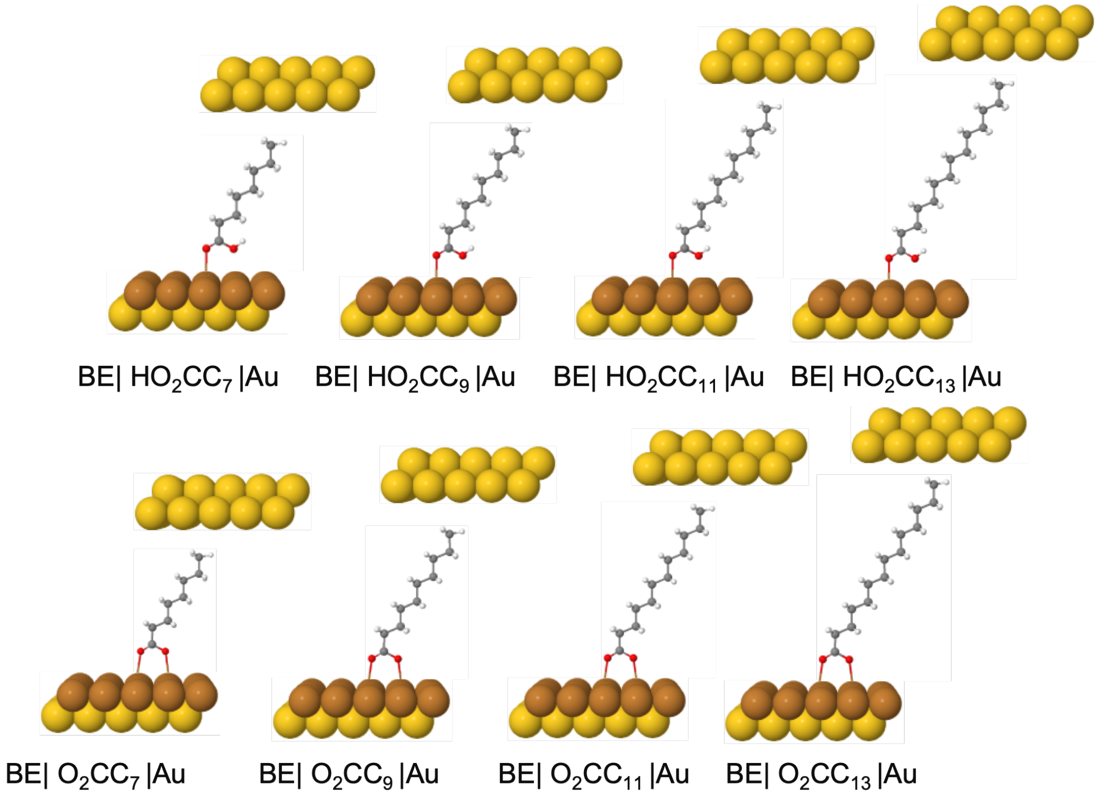

Figure S9. Molecular structures of alkane-carboxylic wires sandwiched between two electrodes $BE/O_2CC_{n-1}//Au$, across a range of values for n (n = 8, 10, 12, 14).

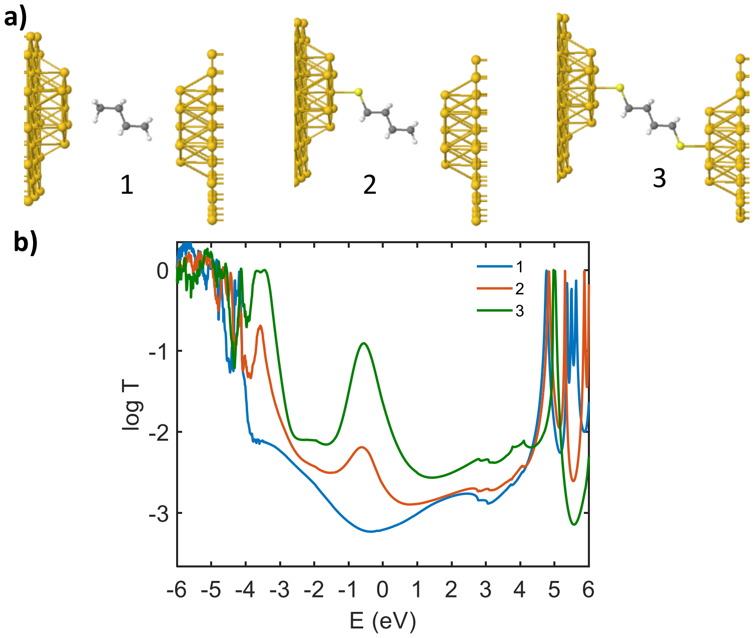

Figure S10. The effect of sulfur GWO on transport through alkanes. (a) Molecular structures of Au//C_4//Au, Au/SC_4//Au and Au/SC_4S/Au. (b) Corresponding transmission coefficient for structures in (a).

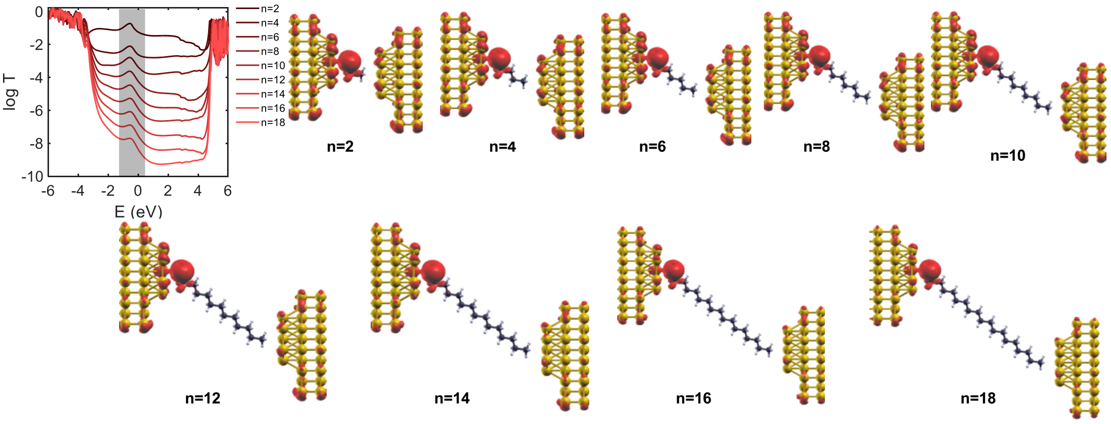

Figure S11. Local density of state iso surfaces for Au/SC_n//Au junctions calculated around the GWO resonances shown by grey area in *T(E)* plot.

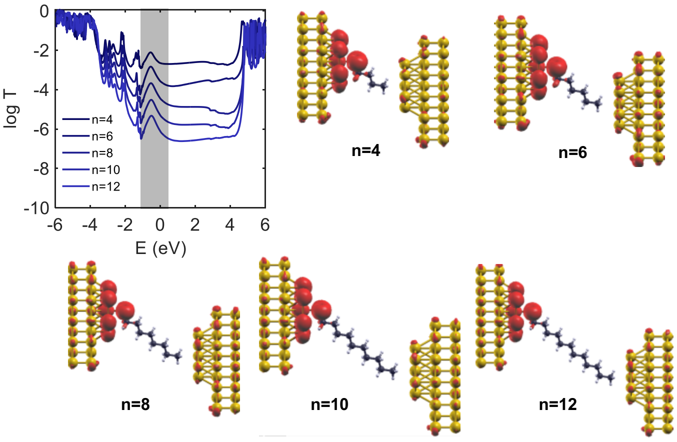

Figure S12. Local density of state iso surfaces for BE/SC_n//Au junctions calculated around the GWO resonances shown by grey area in *T(E)* plot.

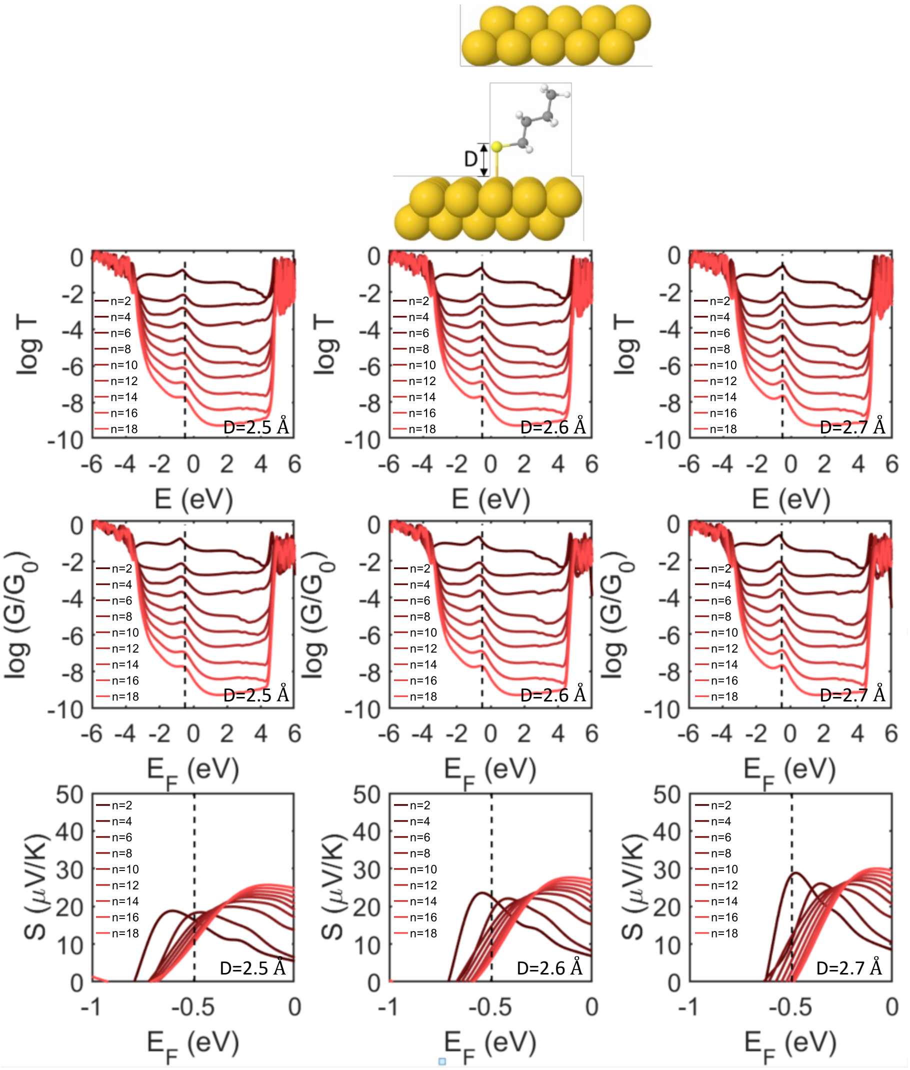

Figure S13. DFT calculated transmission coefficients *T*(*E*), electrical conductance *G* and Seebeck coefficient *S* of Au/SC_n//Au, across a range of values for n (n = 2, ..., 18) with different Au-S distance D = 2.5Å, D = 2.6Å, and D = 2.7Å.

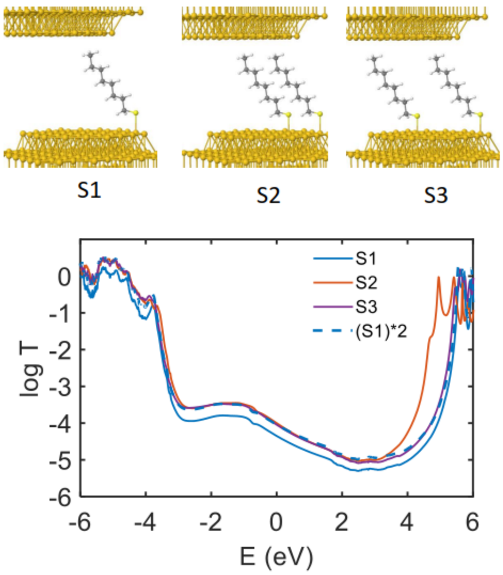

Figure S14. Transmission coefficient of junctions formed by one and two molecules between electrodes. The distance between the molecules is larger in the **S3** configuration compared to the **S2** one.

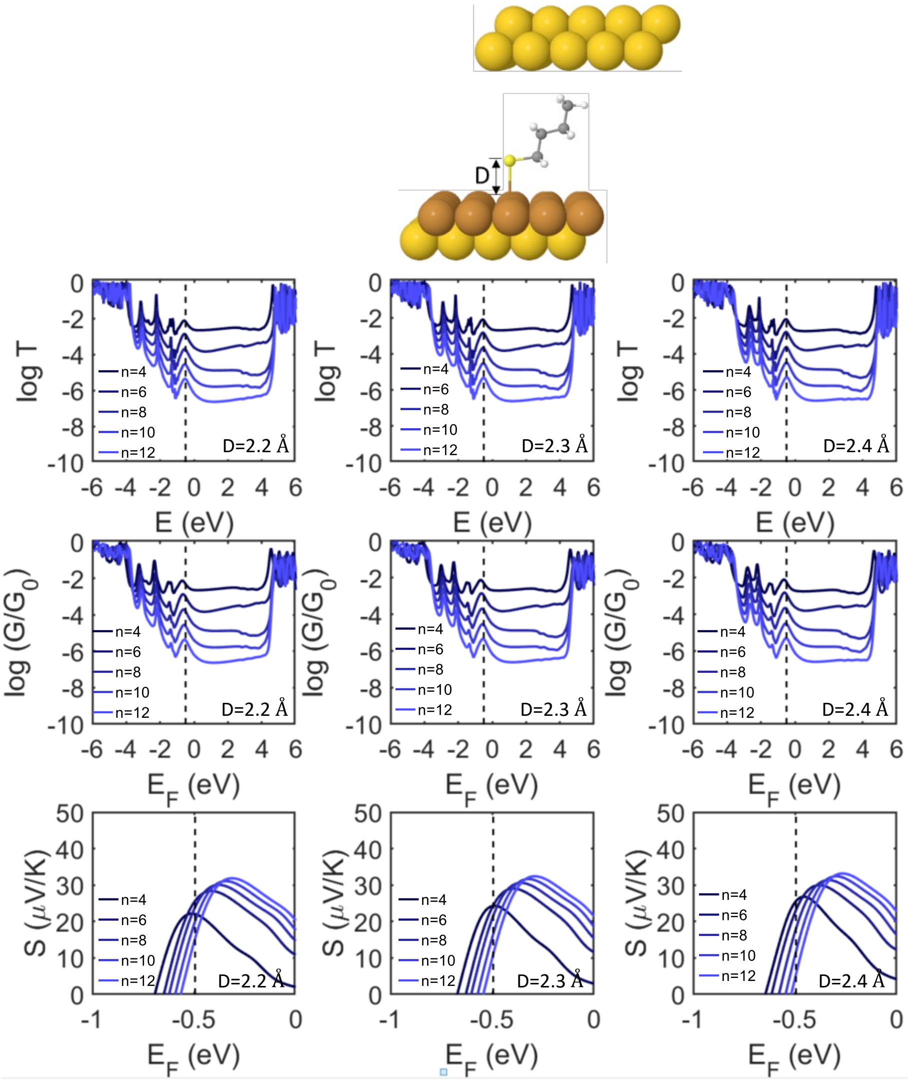

Figure S15. DFT calculated transmission coefficients *T*(*E*), electrical conductance *G* and Seebeck coefficient *S* of BE/SC$_n$//Au, across a range of values for n (n = 4, ..., 12) with different Cu-S distance D = 2.2Å, D = 2.3Å, and D = 2.4Å.

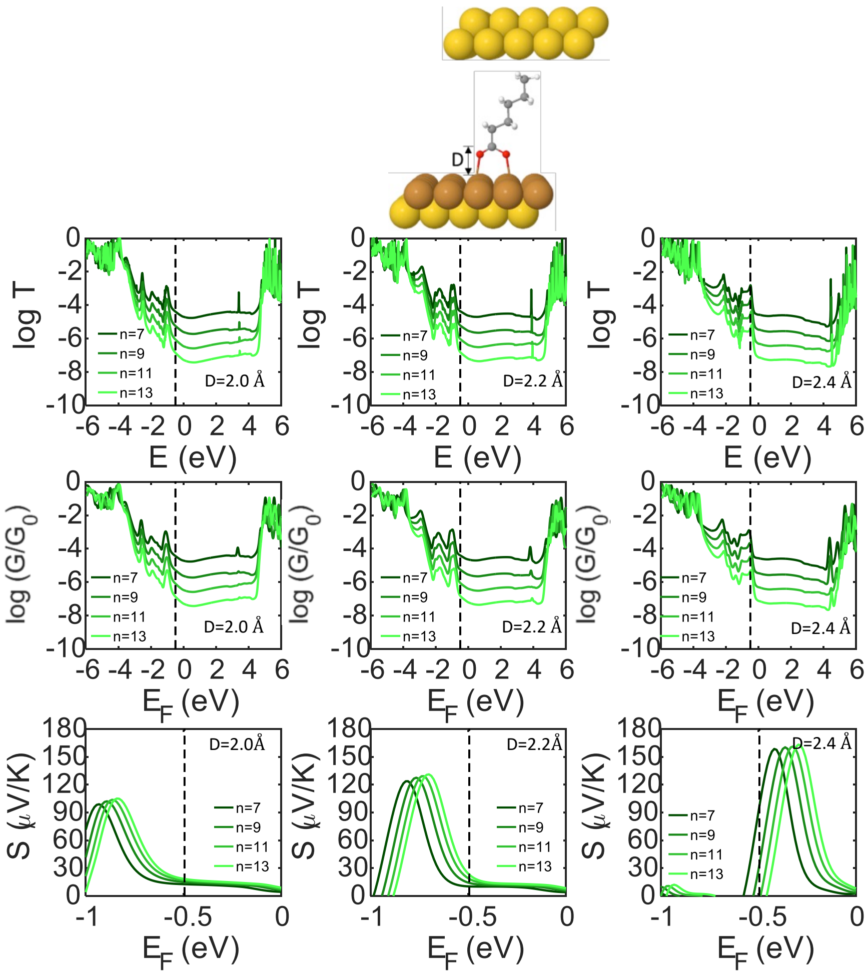

Figure S16. DFT calculated transmission coefficients $T(E)$, electrical conductance G and Seebeck coefficient S of BE/O_2CC_{n-1}//Au, across a range of values for n (n = 8, 10, 12, 14) with different Cu-C distance D = 2.0Å, D = 2.2Å, and D = 2.4Å.

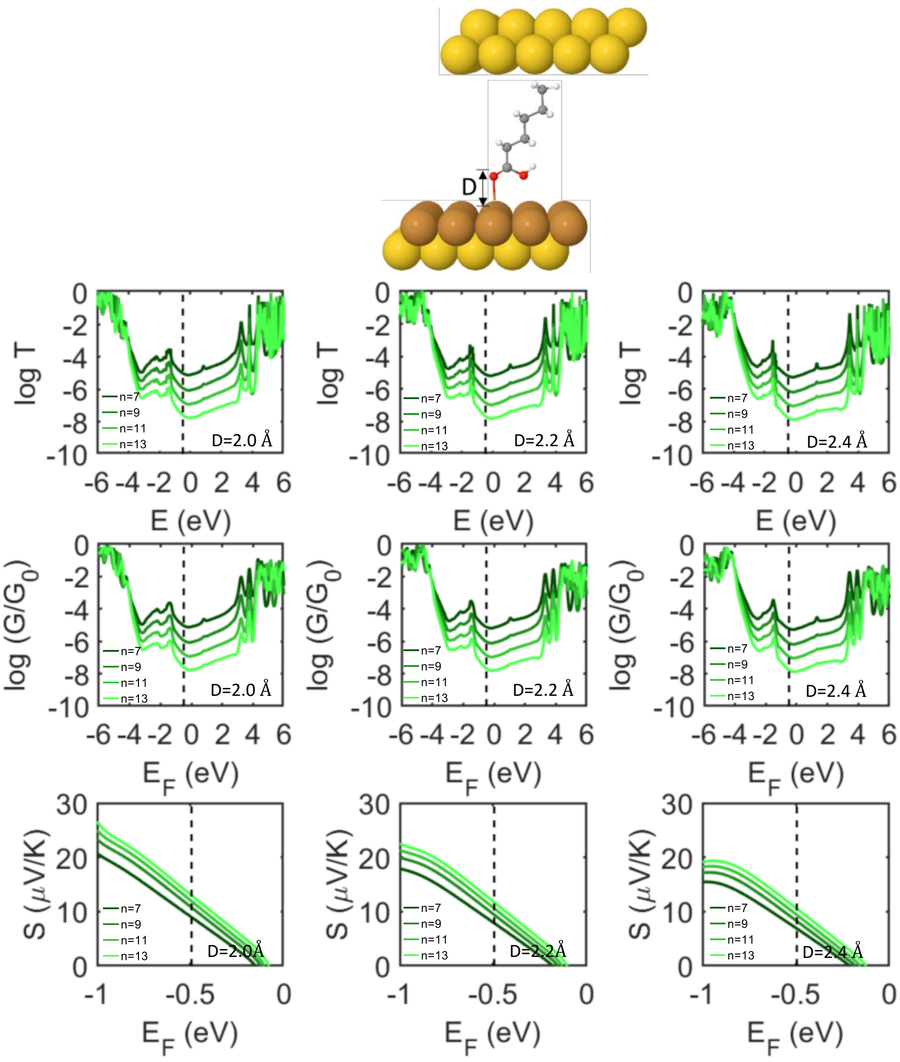

Figure S17. DFT calculated transmission coefficients *T*(*E*), electrical conductance *G* and Seebeck coefficient *S* of BE/HO_2CC_{n-1}//Au, across a range of values for n (n = 8, 10, 12, 14) with different Cu-C distance D = 2.0Å, D = 2.2Å, and D = 2.4Å.

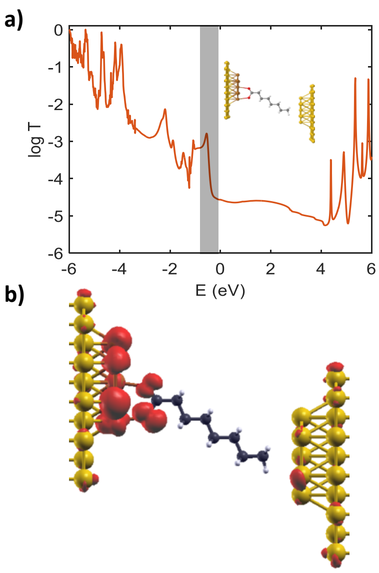

Figure S18. (a) Transmission coefficient for alkane-carboxylic wires sandwiched between two electrodes (the two oxygen atoms bind to the Cu layer), (b) Local density of state iso surfaces for BE/O_2CC_7//Au junction calculated around the GWO resonances shown by grey area in a.

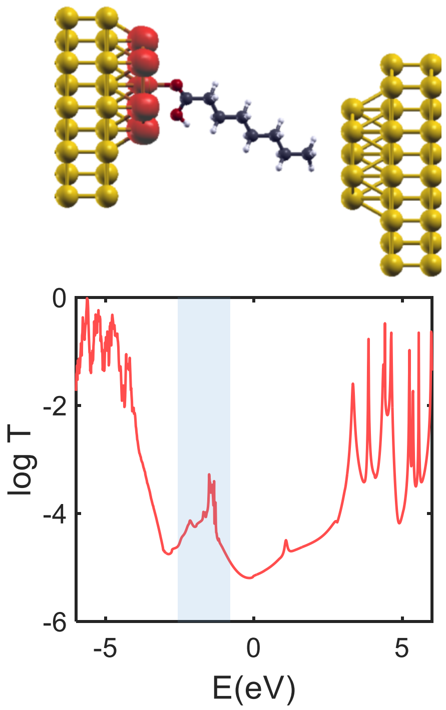

Figure S19. Local density of state calculation for energies around the GWO state (light blue area) for an alkane chain with the -COOH connected to Cu layer via one oxygen atom.

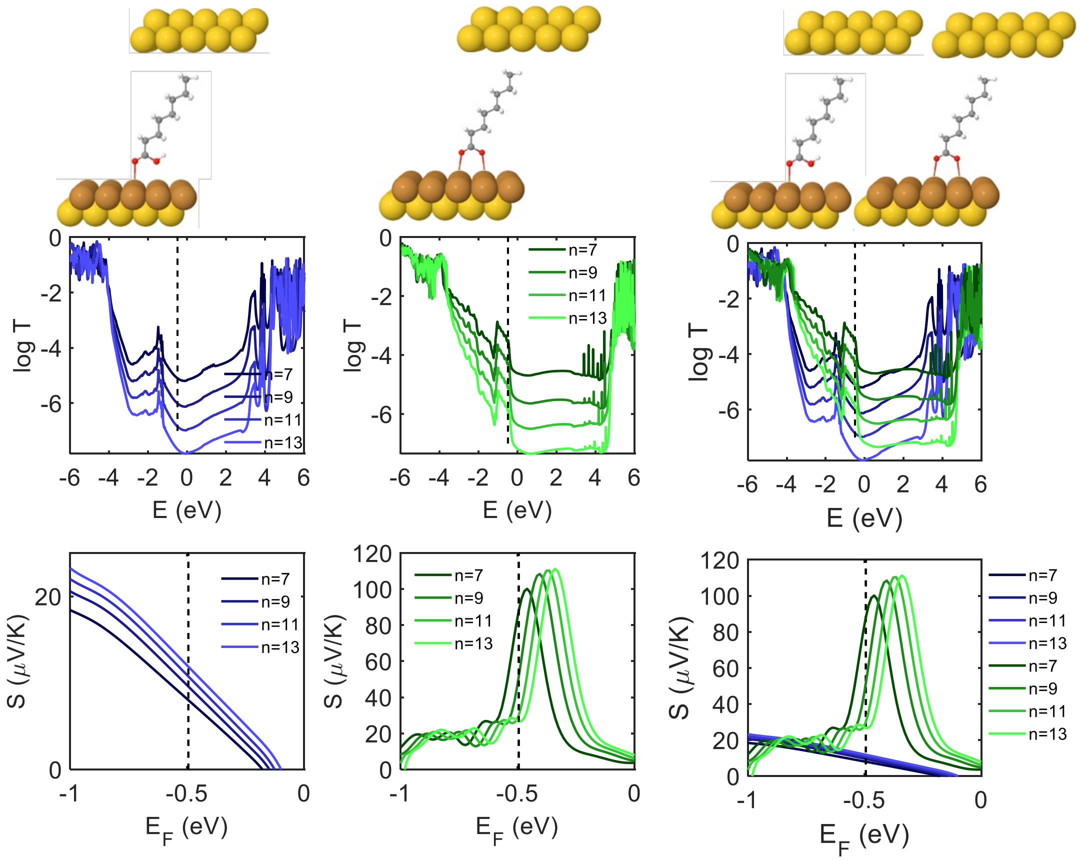

Figure S20. The effect of contacting modalities between -COOH and Cu adlayer.

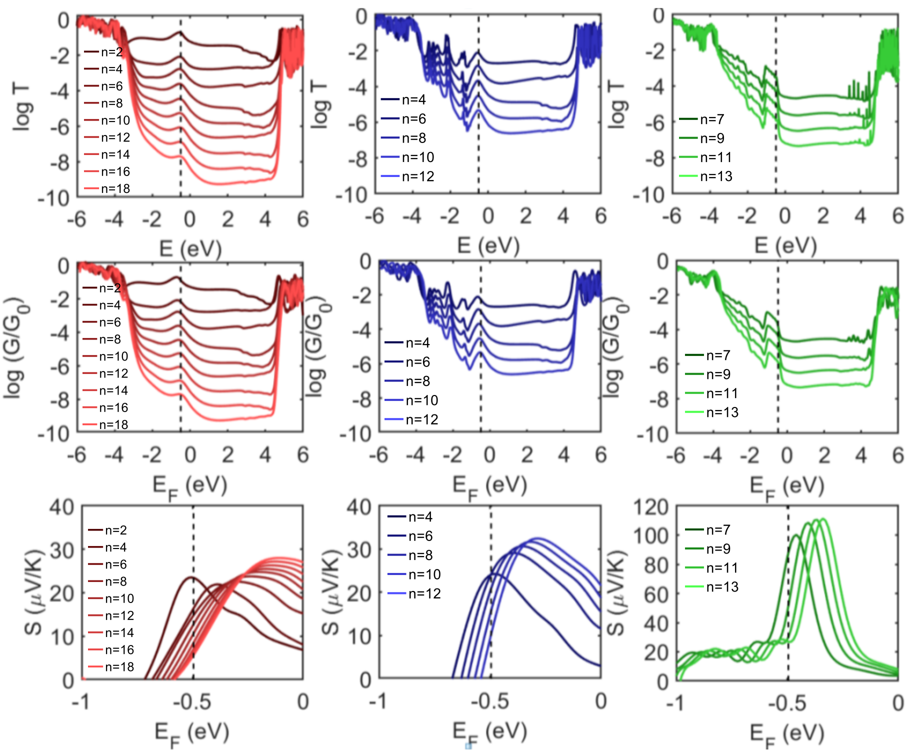

Figure S21. Thermoelectric properties of alkane molecular junctions. DFT calculated transmission coefficients *T*(*E*), electrical conductance *G* and average Seebeck coefficient of Au/SC_n//Au (red), BE/SC_n//Au (blue) and BE/O_2CC_{n-1}//Au (green).

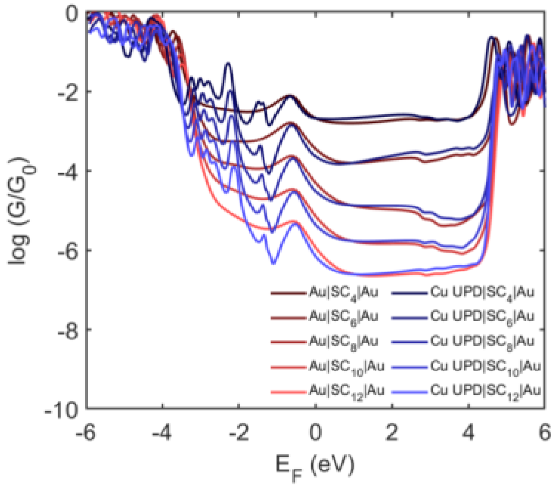

Figure S22. Transport through alkanes with -SH between gold electrodes with and without Cu adlayer.

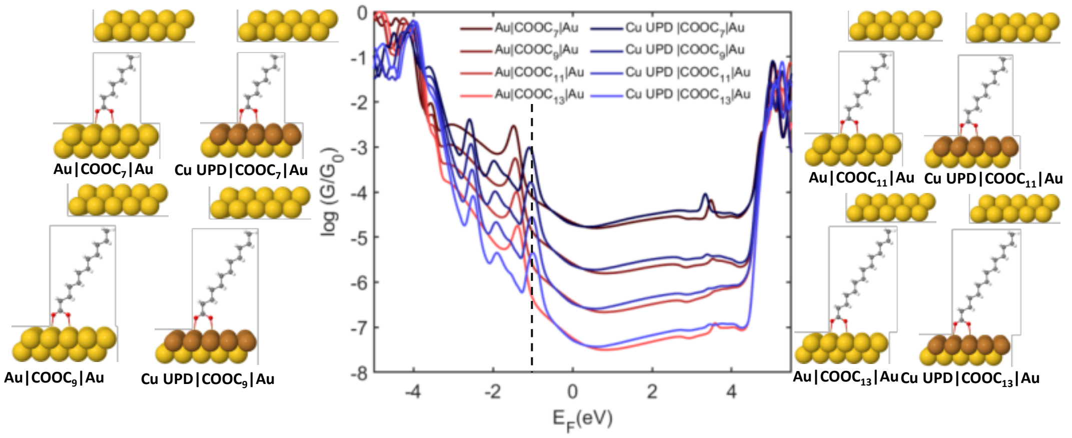

Figure S23. Transport through alkanes with -COOH between gold electrodes with and without Cu adlayer.

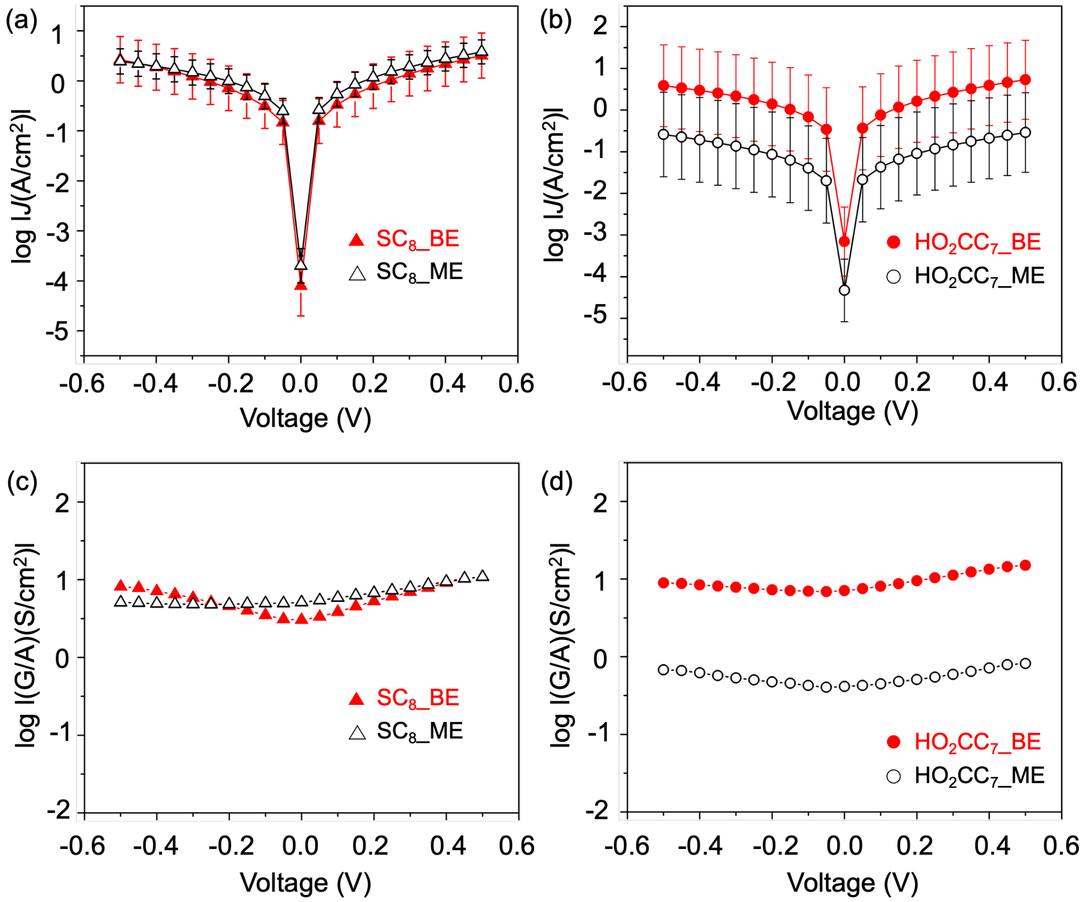

Figure S24. Log|J|-V curves of (a) HSC_8 and (b) HO_2CC_7 and log|G/A|-V curves of (c) HSC_8 and (d) HO_2CC_7 on BE and ME, respectively.

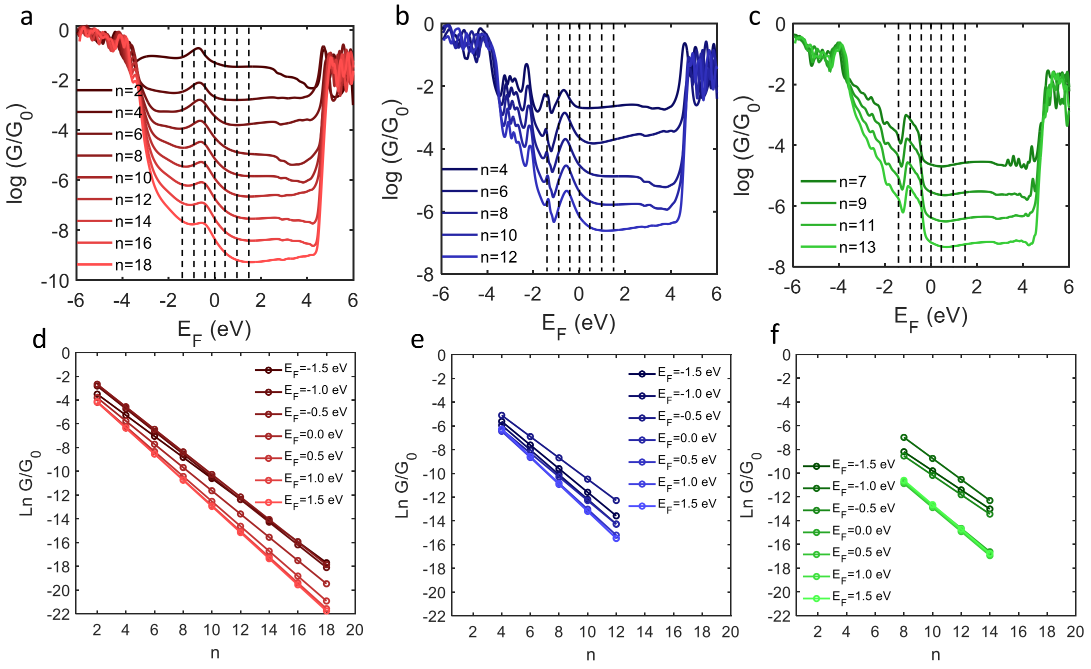

Figure S25. Room temperature electrical conductance as a function of electrodes Fermi energy for Au/SC_n//Au (red), BE/SC_n//Au (blue) and BE/O_2CC_{n-1}//Au (green) (a,b,c). Calculated natural logarithm of conductance (beta factor) versus different Fermi energies (d,e,f) show on by dashed lines in a-c.

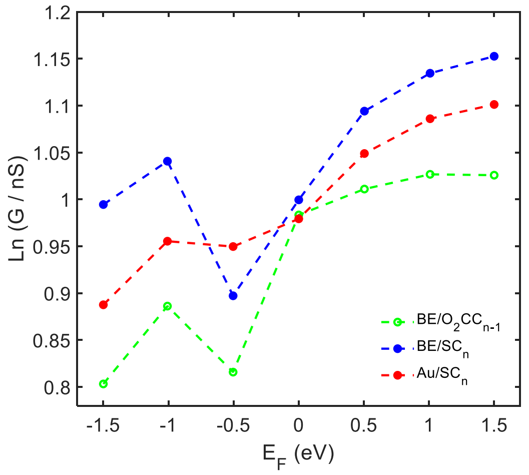

Figure S26. Beta factor versus Fermi energy for (Au/SC_n//Au), (BE/SC_n//Au) and (BE/O_2CC_{n-1}//Au).

Table S1. Summary of data of thermoelectric junction measurements of HO_2CC_{n-1} on bimetallic electrode.

n in HO_2CC_{n-1}	ΔT(K)	Samples	Junctions	Counts	$\Delta V_{mean} \pm \sigma\Delta V$	S (μV/K)	Yield (%)
	4	2	34	2550	-96 ± 18		82
	8	2	34	2550	-132 ± 23		88
8	12	2	34	2550	-189 ± 20	13.3 ± 0.6	88
	15	2	34	2550	-227 ± 24		82
	20	2	28	2100	-295 ± 36		79
	4	2	36	2700	-43 ± 5		89
	8	2	40	3000	-77 ± 7		90
10	12	2	41	3075	-106 ± 12	8.9 ± 0.6	88
	15	2	31	2325	-133 ± 20		77
	20	2	33	2475	-168 ± 16		85
	4	2	22	1650	-39 ± 8		100
	8	2	28	2100	-58 ± 15		93
12	12	2	24	1800	-81 ± 24	7.0 ± 0.4	92
	15	2	32	2400	-110 ± 20		94
	20	2	29	2175	-135 ± 27		90
	4	2	25	1875	-40 ± 9		96
	8	2	25	1875	-65 ± 10		92
14	12	2	24	1800	-81 ± 7	6.4± 0.4	100
	15	2	22	1650	-100 ± 12		91
	20	2	23	1725	-134 ± 18		78

Table S2. Summary of data of thermoelectric junction measurements of HSC_n on bimetallic electrode.

n in HSC_n	ΔT(K)	Samples	Junctions	Counts	ΔVmean ± σΔV	*S* (μV/K)	Yield (%)
4	4	2	34	2550	-63 ± 11	8.6 ± 0.4	88
	8	2	29	2175	-91 ± 10		90
	12	2	34	2550	-129 ± 23		88
	15	2	32	2400	-152 ± 13		88
	20	2	36	2700	-178 ± 18		83
6	4	2	30	2250	-39 ± 6	7.9 ± 0.3	87
	8	2	34	2550	-62 ± 9		94
	12	2	21	1575	-91 ± 16		86
	15	2	26	1950	-113 ± 18		92
	20	2	28	2100	-152 ± 16		86
8	4	2	20	1500	-45 ± 6	6.9 ± 0.2	90
	8	2	20	1500	-71 ± 9		90
	12	2	21	1575	-93 ± 7		95
	15	2	22	1650	-114 ± 16		91
	20	2	23	1725	-136 ± 14		87
10	4	2	24	1800	-31 ± 10	5.7 ± 0.3	100
	8	2	26	1950	-49 ± 13		92
	12	2	28	2100	-64 ± 12		100
	15	2	32	2400	-83 ± 15		100
	20	2	26	1950	-110 ± 21		92
12	4	2	21	1575	-16 ± 3	5.0 ± 0.1	95
	8	2	24	1800	-31 ± 5		100
	12	2	24	1800	-50 ± 14		100
	15	2	18	1350	-63 ± 10		100
	20	2	20	1500	-79 ± 5		90

Table S3. Summary of reported Seebeck coefficient of HO_2CC_{n-1} on Ag^{TS}.[20]

n in HO_2CC_{n-1}	*S* (μV/K)
4	5.2 ± 0.3
6	4.1 ± 0.2
8	3.1 ± 0.2

Table S4. Summary of reported Seebeck coefficient of HSC_n on Au^{TS}.[21]

n in HSC_n	S (μV/K)
4	6.4 ± 0.7
6	5.1 ± 0.6
8	3.5 ± 0.2
10	3.3 ± 0.1
12	3.0 ± 0.1
14	2.9 ± 0.2

Table S5. Frontier orbitals of alkane wires (HSC_4), (HSC_4SH) and (C_4).

Molecule	HOMO-3	HOMO-2	HOMO-1	HOMO	Gap	LUMO	LUMO+1	LUMO+2	LUMO+3
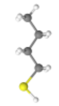	-7.76 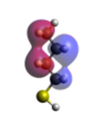	-7.61 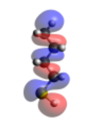	-7.37 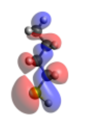	-4.74 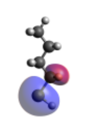	4.98	0.24 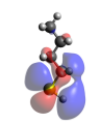	1.69 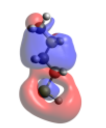	2.19 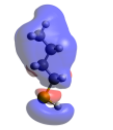	2.79 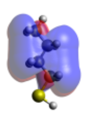
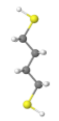	-7.50 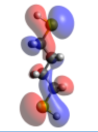	-7.46 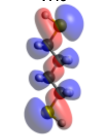	-4.83 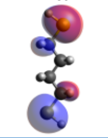	-4.82 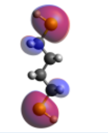	4.92	0.10 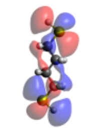	0.20 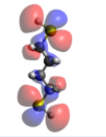	1.54 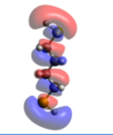	1.61 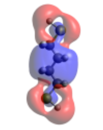
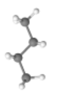	-8.14 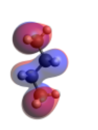	-7.85 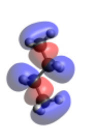	-7.49 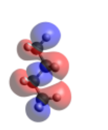	-7.25 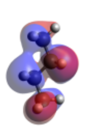	9.46	2.21 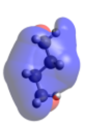	2.78 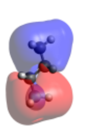	2.93 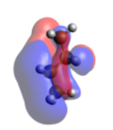	3.06 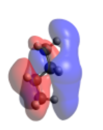

Table S6. Frontier orbitals of alkane wires (HO_2CC_7) and (C_7).

Molecule	HOMO-3	HOMO-2	HOMO-1	HOMO	Gap	LUMO	LUMO+1	LUMO+2	LUMO+3
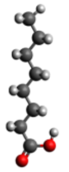	-7.67 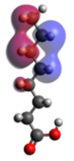	-7.39 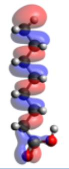	-6.79 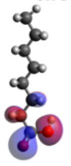	-5.38 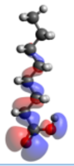	5.09	-0.29 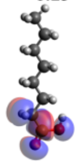	1.37 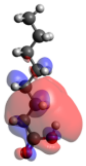	1.69 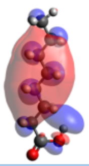	2.00 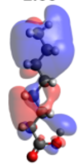
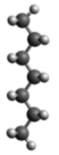	-7.50 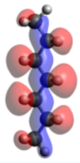	-7.45 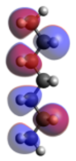	-7.39 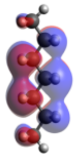	-6.92 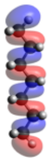	8.98	2.05 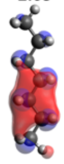	2.20 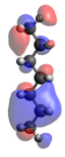	2.60 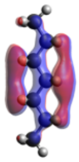	2.70 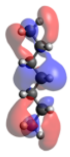

Table S7. Summary of effects of electrode materials for HO_2CC_7 and HSC_8 on current density and conductance.

SAMs	# of samples	# of working junctions	yields (%)	$\log(J(+0.5\ V))_{mean} \pm \sigma_{\log J}$ (A/cm^2)	$\log(G/A$(at 0 V)) (S/cm^2)
HO_2CC_7/BE[a]	2	18	90	0.73±0.94	0.85
HSC_8/BE[a]	2	16	100	0.51±0.46	0.48
HO_2CC_7/ME[b]	2	18	94	-0.54±0.95	-0.38
HSC_8/ME[c]	2	18	89	0.58±0.24	0.71

[a]Cu/Au^{TS}; [b]Ag^{TS}; [c]Au^{TS}

3. References

(1) Chen, I. W. P.; Chen, C.-C.; Lin, S.-Y.; Chen, C.-h. Effect of Underpotentially Deposited Adlayers on Sulfur Bonding Schemes of Organothiols Self-Assembled on Polycrystalline Gold: sp or sp3 Hybridization. *J. Phys. Chem. B* **2004**, *108* (45), 17497-17504. DOI: 10.1021/jp047495p

(2) Jennings, G. K.; Laibinis, P. E. Self-Assembled n-Alkanethiolate Monolayers on Underpotentially Deposited Adlayers of Silver and Copper on Gold. *J. Am. Chem. Soc.* **1997**, *119* (22), 5208-5214. DOI: 10.1021/ja962973y

(3) Ross, M. B.; Dinh, C. T.; Li, Y.; Kim, D.; De Luna, P.; Sargent, E. H.; Yang, P. Tunable Cu Enrichment Enables Designer Syngas Electrosynthesis from CO(2). *J. Am. Chem. Soc.* **2017**, *139* (27), 9359-9363. DOI: 10.1021/jacs.7b04892

(4) Xu, J.; Li, R.; Xu, C.-Q.; Zeng, R.; Jiang, Z.; Mei, B.; Li, J.; Meng, D.; Chen, J. Underpotential-deposition synthesis and in-line electrochemical analysis of single-atom copper electrocatalysts. *Appl. Catal. B Environ.* **2021**, *289*. DOI: 10.1016/j.apcatb.2021.120028

(5) Lin, S.-Y.; Chen, C.-h.; Chan, Y.-C.; Lin, C.-M.; Chen, H.-W. Self-Assembly of Alkanoic Acids on Gold Surfaces Modified by Underpotential Deposition. *J. Phys. Chem. B* **2001**, *105* (21), 4951-4955. DOI: 10.1021/jp004329i

(6) Jang, J.; He, P.; Yoon, H. J. Molecular Thermoelectricity in EGaIn-Based Molecular Junctions. *Acc. Chem. Res.* **2023**, *56* (12), 1613-1622. DOI: 10.1021/acs.accounts.3c00168

(7) Chiechi, R. C.; Weiss, E. A.; Dickey, M. D.; Whitesides, G. M. Eutectic gallium-indium (EGaIn): a moldable liquid metal for electrical characterization of self-assembled monolayers. *Angew. Chem., Int. Ed.* **2008**, *47* (1), 142-144. DOI: 10.1002/anie.200703642

(8) Park, S.; Yoon, H. J. New Approach for Large-Area Thermoelectric Junctions with a Liquid Eutectic Gallium-Indium Electrode. *Nano Lett.* **2018**, *18* (12), 7715-7718. DOI: 10.1021/acs.nanolett.8b03404

(9) Soler, J. M.; Artacho, E.; Gale, J. D.; García, A.; Junquera, J.; Ordejón, P.; Sánchez-Portal, D. The SIESTA method forab initioorder-Nmaterials simulation. *J. Phys.: Condens. Matter.* **2002**, *14* (11), 2745-2779. DOI: 10.1088/0953-8984/14/11/302

(10) Ferrer, J.; Lambert, C. J.; García-Suárez, V. M.; Manrique, D. Z.; Visontai, D.; Oroszlany, L.; Rodríguez-Ferradás, R.; Grace, I.; Bailey, S. W. D.; Gillemot, K.; et al. GOLLUM: a next-generation simulation tool for electron, thermal and spin transport. *New J. Phys.* **2014**, *16* (9). DOI: 10.1088/1367-2630/16/9/093029

(11) Sadeghi, H. Theory of electron, phonon and spin transport in nanoscale quantum devices. *Nanotechnology* **2018**, *29* (37), 373001. DOI: 10.1088/1361-6528/aace21

(12) Weiss, E. A.; Kaufman, G. K.; Kriebel, J. K.; Li, Z.; Schalek, R.; Whitesides, G. M. Si/SiO2-templated formation of ultraflat metal surfaces on glass, polymer, and solder supports: their use as substrates for self-assembled monolayers. *Langmuir* **2007**, *23* (19), 9686-9694. DOI: 10.1021/la701919r

(13) Tao, Y. T. Structural comparison of self-assembled monolayers of n-alkanoic acids on the surfaces of silver, copper, and aluminum. *J. Am. Chem. Soc.* **1993**, *115* (10),

4350-4358. DOI: 10.1021/ja00063a062
(14) Lin, S.-Y.; Tsai, T.-K.; Lin, C.-M.; Chen, C.-h.; Chan, Y.-C.; Chen, H.-W. Structures of Self-Assembled Monolayers of n-Alkanoic Acids on Gold Surfaces Modified by Underpotential Deposition of Silver and Copper: Odd−Even Effect. *Langmuir* **2002**, *18* (14), 5473-5478. DOI: 10.1021/la0157364
(15) Baker, M. V.; Jennings, G. K.; Laibinis, P. E. Underpotentially Deposited Copper Promotes Self-Assembly of Alkanephosphonate Monolayers on Gold Substrates. *Langmuir* **2000**, *16* (7), 3288-3293. DOI: 10.1021/la991247g
(16) Jang, J.; Kong, G. D.; Kang, H.; Yoon, H. J. Implication of Current–Voltage Curve Shape in Molecular Electronics. *J. Phys. Chem. C* **2023**, *127* (12), 6025-6033. DOI: 10.1021/acs.jpcc.2c09086
(17) Park, J.; Kodaimati, M. S.; Belding, L.; Root, S. E.; Schatz, G. C.; Whitesides, G. M. Controlled Hysteresis of Conductance in Molecular Tunneling Junctions. *ACS Nano* **2022**, *16* (3), 4206-4216. DOI: 10.1021/acsnano.1c10155
(18) Park, S.; Kang, S.; Yoon, H. J. Power Factor of One Molecule Thick Films and Length Dependence. *ACS Cent. Sci.* **2019**, *5* (12), 1975-1982. DOI: 10.1021/acscentsci.9b01042
(19) Gu, M. W.; Peng, H. H.; Chen, I. P.; Chen, C. H. Tuning surface d bands with bimetallic electrodes to facilitate electron transport across molecular junctions. *Nat. Mater.* **2021**, *20* (5), 658-664. DOI: 10.1038/s41563-020-00876-2
(20) Park, S.; Jang, J.; Yoon, H. J. Validating the Mott Formula with Self-Assembled Monolayer (SAM)-Based Large-Area Junctions: Effect of Length, Backbone, Spacer, Substituent, and Electrode on the Thermopower of SAMs. *J. Phys. Chem. C* **2021**, *125* (36), 20035-20047. DOI: 10.1021/acs.jpcc.1c05623
(21) Park, S.; Cho, N.; Yoon, H. J. Two different length-dependent regimes in thermoelectric large-area junctions of n-alkanethiolates. *Chem. Mater.* **2019**, *31* (15), 5973-5980. DOI: 10.1021/acs.chemmater.9b02461
